# A novel physiological function of pheromone biosynthesis-activating neuropeptide in production of aggregation pheromone

**DOI:** 10.1038/s41598-023-32833-9

**Published:** 2023-04-05

**Authors:** Falguni Khan, Keono Kim, Jeehye Sung, Hangah Lim, Sang-Gyu Kim, Man-Yeon Choi, Yonggyun Kim

**Affiliations:** 1 Department of Plant Medicals, College of Life Sciences, Andong National University, Andong, 36720 Korea; 2 Department of Food Life Science, College of Life Sciences, Andong National University, Andong, 36720 Korea; 3 Department of Biological Sciences, KAIST, Daejon, 34141 Korea; 4 Horticultural Crops Research Unit, USADA-ARS, Corvalis, OR 97330 USA

**Keywords:** Chemical biology, Physiology, Zoology

## Abstract

The western flower thrips, *Frankliniella occidentalis*, is an insect pest, and its aggregation pheromone (AP) plays a crucial role in the recruitment of both sexes. A novel pheromone biosynthesis-activating neuropeptide (PBAN)-like gene is encoded in *F. occidentalis* genome, but its physiological function has yet to be elucidated. This study hypothesized the physiological role played by PBAN in mediating AP production. AP has been known to be produced only by male adults in *F. occidentalis*. Surprisingly, our extraction of headspace volatiles contained two AP components in females as well as in males with similar composition. PBAN injection elevated the AP production whereas RNA interference (RNAi) of the gene expression suppressed the AP production in both sexes. A biosynthetic pathway to produce AP components were predicted and the enzymes catalyzing the main steps were confirmed in their expressions. Individual RNAi treatments of these genes significantly suppressed AP production. RNAi of PBAN gene downregulated the expressions of these biosynthesis-associated genes in both sexes. These results suggest that the novel neuropeptide acts as PBAN mediating AP production through stimulating its biosynthetic machinery in *F. occidentalis*.

## Introduction

The western flower thrips, *Frankliniella occidentalis*, is highly invasive to a wide range of host plants, including high-value crops^1^. It inflicts feeding and ovipositing damages to crops and can indirectly transmit a plant disease virus called tomato spotted wilt virus (TSWV)^2^. To control thrips, different chemical insecticides have been applied to various crops, including hot peppers in Korea^3^. However, several outbreaks of *F. occidentalis* occur every year, and these seriously affect the crop yields^4^. The difficulties involved in effectively controlling this insect pest include its rapid development of insecticide resistance and its behavioral escape from exposure to insecticides^5,6^.

As an alternative control technology, Sampson and Kirk^7^ proposed mass-trapping or other behavioral modification using an aggregation pheromone of *F. occidentalis*. The presence of the aggregation pheromone was proposed by an observation through olfactometer bioassay, which showed an attracting behavior of young virgin females to the odor of adult males, but not to the odor of females^8^. The male odor was chemically assessed, and its two major components were identified as (*R*)-lavandulyl acetate (LA) and neryl (*S*)-2-methylbutanoate (NMB)^9^. Other thrips also produce aggregation pheromone components that are similar to those that have been identified in different thrips species. A congener species, *F*. *intonsa*, has been shown to produce a different ratio of LA and NMB compared to the aggregation pheromone composition of *F. occidentalis*^10^. Another pheromone component, (*R*)-lavandulyl-3-methyl-3-butenoate (LMB), was identified in *Thrips palmi*^11^. Niassy et al.^12^ identified LMB and (R)-lavandulol as the aggregation pheromone of *Megalurothrips sjostedti*. The other type of terpenoid, (2E,6E)-farnesyl acetate, was discovered as the aggregation pheromone of *M. usitatus*^13^*.* In these discoveries of aggregation pheromones of thrips related to *F. occidentalis*, all aggregation pheromones identified in thrips to date are terpenoids, and it is suggested that the aggregation pheromone production in thrips uses a typical terpenoid biosynthetic pathway. However, little was known on the biosynthesis of the aggregation pheromones in thrips.

This study investigated a novel neuropeptide associated with the aggregation pheromone biosynthesis and subsequent biosynthetic pathway of *F. occidentalis*. Using RNA interference (RNAi) of a novel pheromone biosynthesis-activating neuropeptides (Fo-PBAN) of *F. occidentalis* and its synthetic peptide, we tested the role of the neuropeptide in aggregation pheromone biosynthesis. Based on the genome information of *F. occidentalis*^14^, all candidate genes of the enzymes associated with terpenoid biosynthesis were obtained and analyzed in their expressions along with adult development and reproduction. Using individual RNAi treatments, the functional associations of the candidate genes with the pheromone biosynthesis were tested. Finally, the physiological function of Fo-PBAN was further supported by examining its stimulating effect on the biosynthetic pathway of the aggregation pheromone.

## Results

### Effect of adult age on mating in *F. occidentalis*

**Figure 1:**
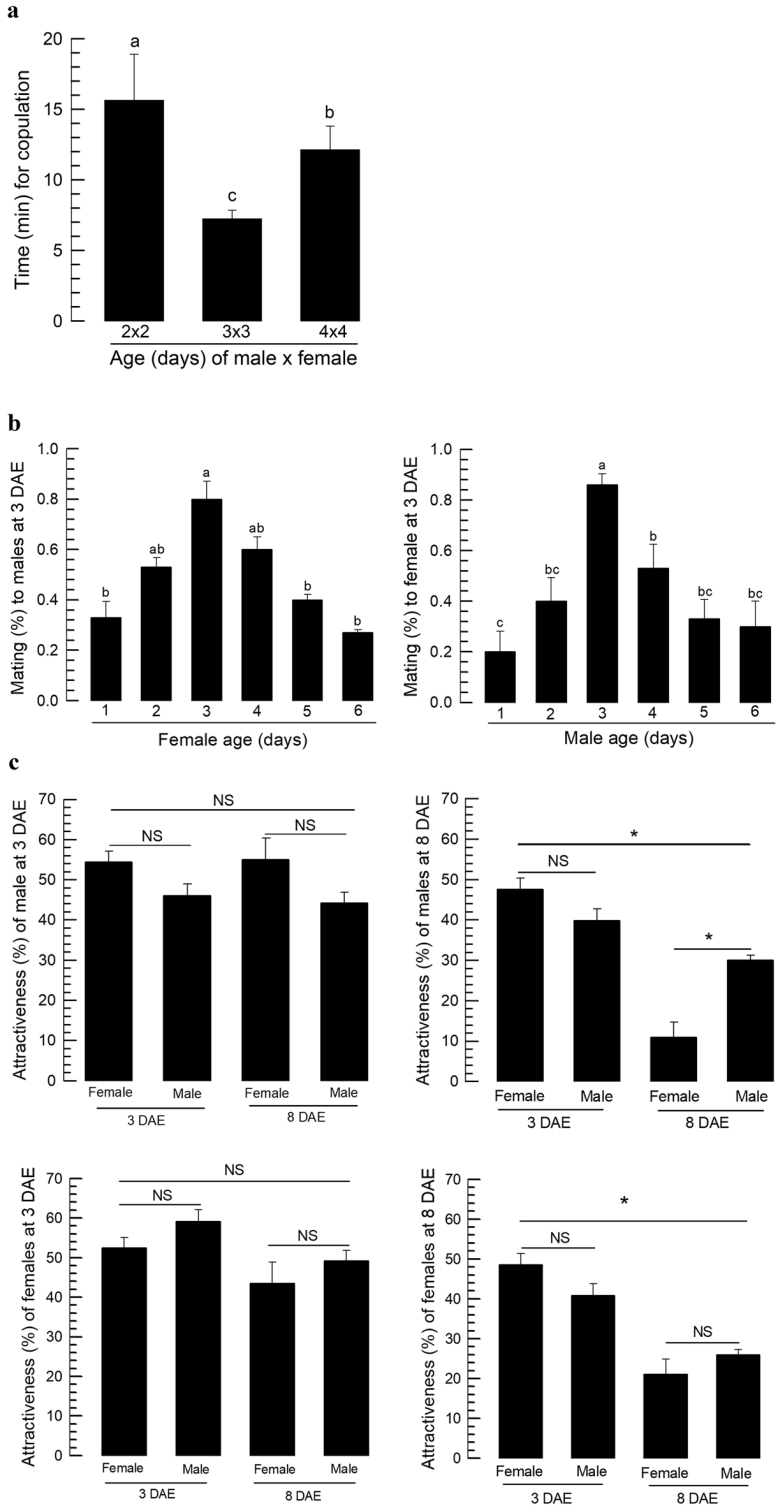
Calling behavior of both male and female adults for mating in *F*. *occidentalis*. (**a**) Variation in the elapsed time to copulate in different adult ages. (**b**) Mating (%) between 3-day-old males and females. (**c**) Choice test based on insect age of males and females. Each trial was replicated three times. Different letters above standard deviation bars indicate significant differences among means at Type I error = 0.05 (LSD test).

Time to copulate was assessed in differently aged couples of *F. occidentalis* (Fig. 1a). Males and females at three days after emergence took only 7 min for copulation, while couples of other ages took more than 10 min. To assess the effect of age on mating, different ages of females were mixed with three-day-old males after adult emergence (Fig. 1b). The males significantly (*P* < 0.05) preferred 2–4-day-old young females for mating. On the other hand, different ages of male adults were mixed with three-day-old females. The females significantly (*P* < 0.05) preferred 3-day-old young males for mating. These results suggest that three-day-old males and females are highly active in terms of mating behavior.

To confirm the age effect on mating with respect to calling behavior, two different age groups were selected: young (3 days old) and old (8 days old) adults (Fig. 1c). Their relative calling behavior was assessed using a Y-tube olfactometer. Young males and females were able to attract both young and old adults in both sexes. However, old males and females significantly lost their attractiveness to old adults. These results support the age effect of *F. occidentalis* on calling other adults and suggested that young adults are active in calling other adults as both males and females presumably by emitting aggregation pheromone.

### Aggregation pheromone production in male and female adults of *F. occidentalis*

**Figure 2:**
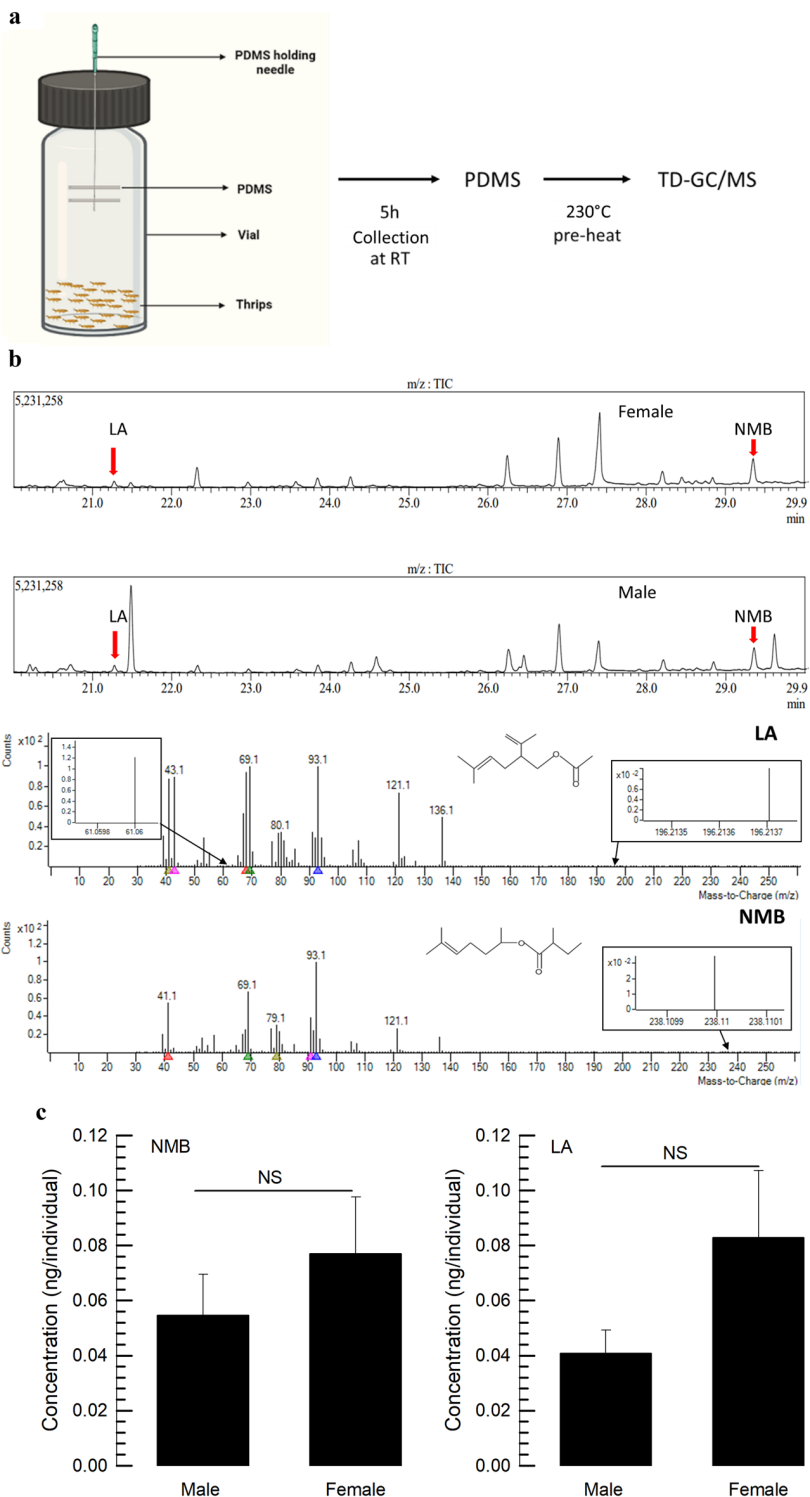
Identification of aggregation pheromone: neryl (S)-2-methylbutanoate (NMB) and lavandulyl acetate (LA) in male and female *F. occidentalis*. (**a**) Schematic diagram of aggregation pheromone collection process in polydimethylsiloxane (PDMS) fiber for GC/MS analysis. (**b**) Representative chromatograms of NMB and LA from females and males. GC–MS chromatograms indicating NMB and LA peaks in arrow. (**c**) NMB and LA concentration released by individual insects calculated for male and female *F. occidentalis*. Approximately 50 individuals < 3 days old male and female thrips were transferred to a 10 mL amber vial. Two PDMS fiber tubes were kept inside this vial for the collection of volatile compounds. Three separate replications were used for each treatment. Different letters above standard deviation bars indicate significant differences among means at Type I error = 0.05 (LSD test).

Headspace volatiles released from the adult thrips (*n* = 50, < 3 days old) were collected using polydimethylsiloxane (PDMS) tubing (Fig. 2a). The collected volatiles in the PDMS were released through thermal desorption unit and assessed by GC–MS. Two components (NMB and LA) of *F. occidentalis* aggregation pheromone were detected in a chromatogram of male volatiles: LA at a retention time of 21.25 min and NMB at a retention time of 29.45 min (Fig. 2b). Surprisingly, the two components were also detected in the chromatogram of the female volatiles. From the GC–MS analysis, the molar ratios of LA:NMB were similar in both sexes, at 1:1.37 in males and 1:1.05 in females. Furthermore, the LA + NMB concentrations released by each individual were ~ 0.09 ng/female and ~ 0.15 ng/male, but not significantly (*P* < 0.05) different between sexes (Fig. 2c).

### Pheromone biosynthesis-activating peptide (PBAN) and its physiological function in production of aggregation pheromone in *F. occidentalis*

**Figure 3:**
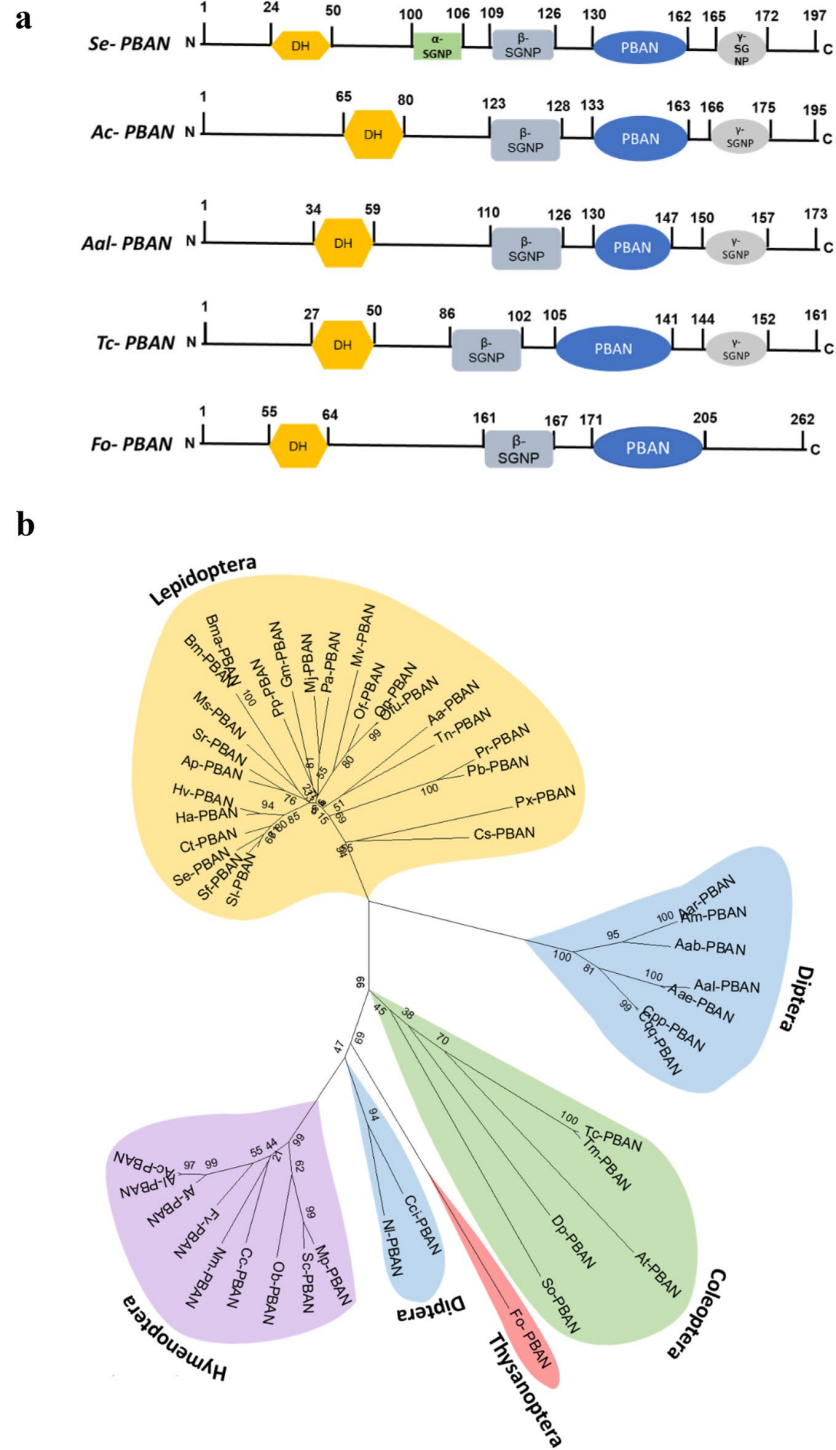
Prediction of the functional domain of *Fo-PBAN*. (**a**) Comparison and alignment of the functional domain with other insects of different orders. *Spodoptera exigua* (Se), *Apis cerana* (Ac), *Aedes albopictus* (Aal), *Tribolium castaneum* (Tc), and *Frankliniella occidentalis* (Fo). (**b**) Phylogenetic relationship of *Fo-PBAN* with PBAN amino acid sequences of other insects. The tree was constructed with the Neighbor-Joining method using MEGA6.06. The bootstrapping values on branches were obtained with 1000 replications. All of the GenBank accession numbers used in this analysis are listed in Table S3. PBAN, pheromone biosynthesis activating neuropeptide.

A putative *PBAN* of *F. occidentalis* (*Fo-PBAN*) encodes an ORF of 262 amino acids (Fig. 3a). The ORF encodes two other neuropeptides (diapause hormone (DH) and β-subesophageal neuropeptide (β-SGNP) in addition to a 34 amino acid length PBAN with the conserved FXPRL sequence at the C terminus (Fig. S1). A phylogenetic analysis with other insect orthologs showed that *Fo-PBAN* shares a close relation with dipteran and hymenopteran PBAN genes (Fig. 3b).

**Figure 4:**
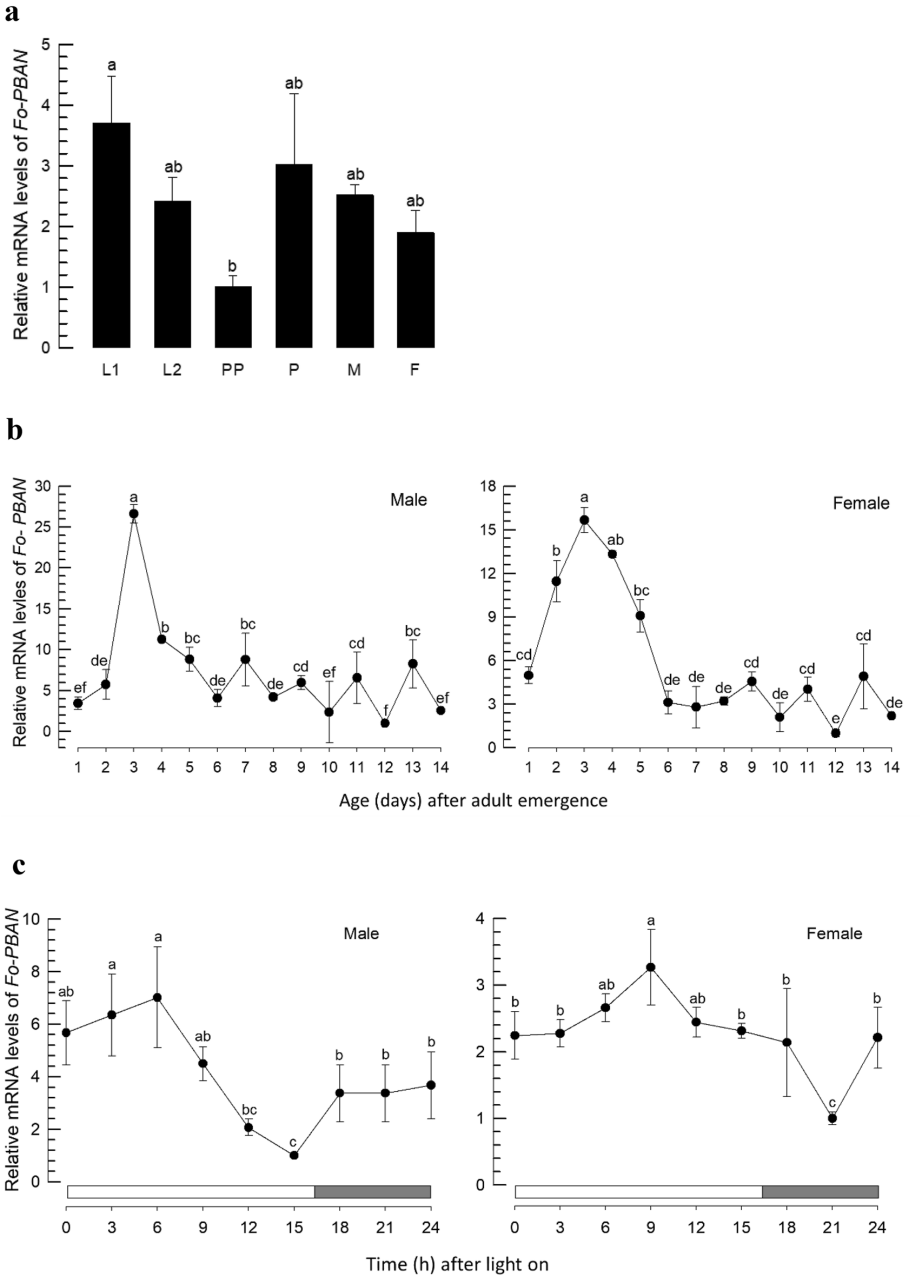
Expression profile of *Fo-PBAN* gene in *F. occidentalis*. (**a**) Expression pattern of *Fo-PBAN* in different developmental stages: larva (L1 and L2), prepupa (PP), pupa (P), male (M) and female (F). (**b**) PBAN mRNA expression levels in male and female for 14 days of *F*. *occidentalis* after emergence from pupa and (**c**) mRNA expression level in male and female *F*. *occidentalis* based on time (h) after light on. An elongation factor, *EF1*, was used as an internal control to normalize the target gene expression level. Three replications were used per treatment. Different letters above standard deviation bars indicate significant differences among means at Type I error = 0.05 (LSD test). PBAN, pheromone biosynthesis activating neuropeptide.

*Fo-PBAN* was expressed in all developmental stages from larva to adult (Fig. 4a). RT-qPCR analysis in adults indicated that the expression levels of *Fo-PBAN* varied among adult ages, where the highest expression levels occurred at 3 days after emergence in both male and female adults (Fig. 4b). The expression levels of *Fo-PBAN* were also shown to fluctuate in a day and exhibited a diel pattern (Fig. 4c).

**Figure 5:**
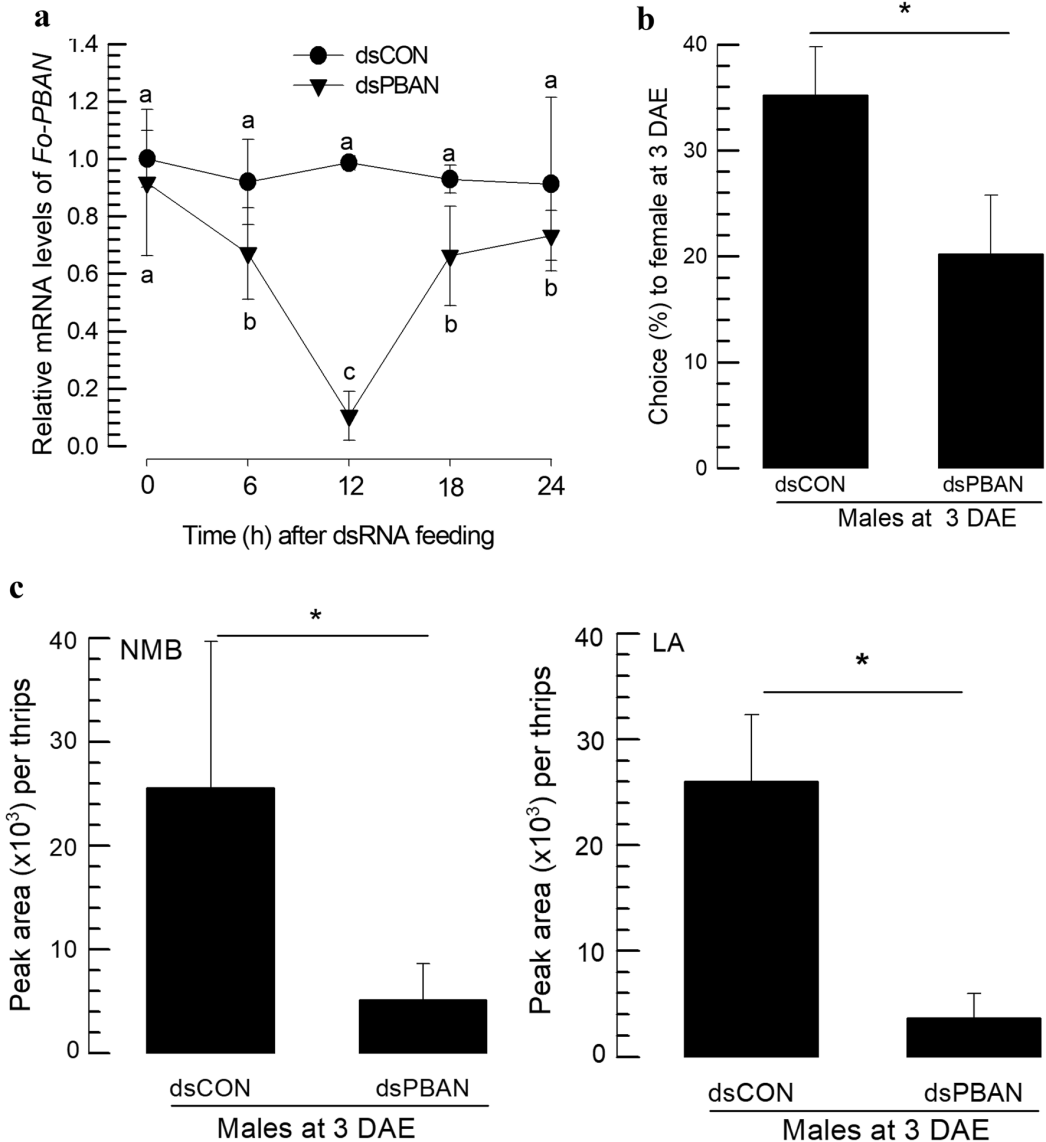
Effect of RNA interference (RNAi) specific to *Fo-PBAN* in *F. occidentalis*. (**a**) Knockdown effect in mRNA expression in male *F. occidentalis* after dsPBAN application. RNAi was performed using the feeding method. A viral gene, *CpBV302*, was used as a control dsRNA (dsCON). An elongation factor, *EF1*, was used to normalize the expression level. For each treatment, three replications were used. (**b**) Response to male by female *F. occidentalis* after knockdown of *Fo-PBAN* in male insects in the Y-tube olfactometer. In the Y-tube olfactometer, the control chamber was kept vacant, and the treatment chamber contained dsRNA-fed male insects. For each replication, approximately 50 individual adults (~ 3 days old) per treatment were used for the Y-tube test. (**c)** PBAN gene-specific dsRNA-fed male thrips were used to analyze neryl (S)-2-methylbutanoate (NMB) and lavandulyl acetate (LA) compound by using GC–MS. Approximately 50 individual male thrips were transferred to a 10 mL amber vial. Two PDMS fiber tubes were placed inside the vial to collect volatile compounds. Both control and dsPBAN-fed insects were considered as treatment, and each treatment was replicated three times. Different letters above standard deviation bars indicate significant differences among means at Type I error = 0.05 (LSD test). PBAN, pheromone biosynthesis activating neuropeptide.

To investigate the function of Fo-PBAN in aggregation pheromone biosynthesis, its gene expression was suppressed by RNAi (Fig. 5). Male adults were fed with dsRNA specific to the gene for 12 h. This dsRNA feeding treatment led to a significant (*P* < 0.05) reduction in the gene expression levels, where Fo-PBAN expression levels were reduced for at least 24 h after dsRNA treatment compared to the control thrips treated with the same amount of non-target dsRNA (= dsCON) (Fig. 5a). The RNAi treatment significantly (*P* < 0.05) impaired the males to attract females (Fig. 5b). SPME coupled with GC–MS of the headspace volatiles showed a significant (*P* < 0.05) reduction among RNAi-treated males compared to dsCON-treated males in terms of both NMB and LA amounts (Fig. 5c).

**Figure 6:**
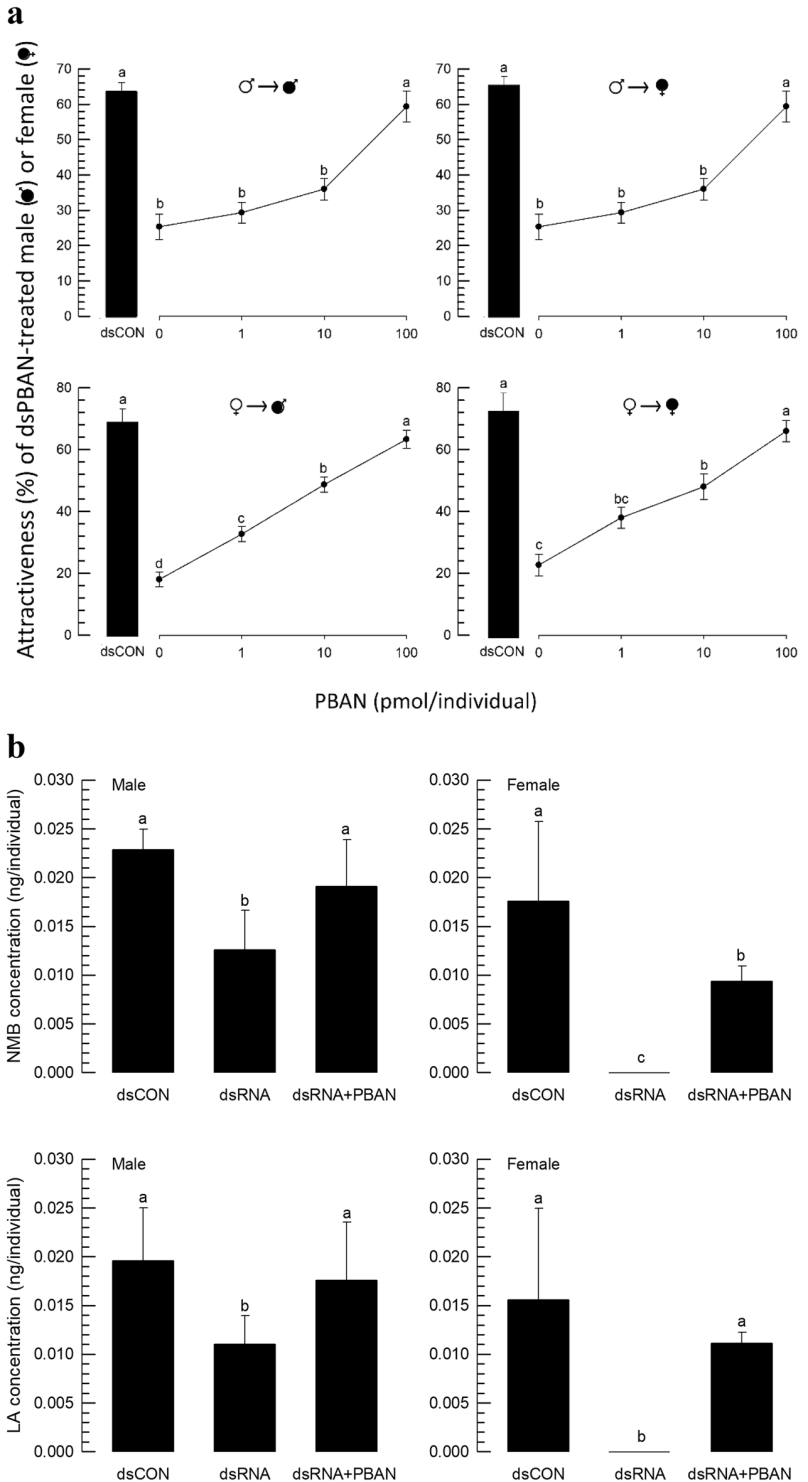
Effect of PBAN injection on *F. occidentalis* treated with dsRNA (dsPBAN) specific to Fo-PBAN. Control (dsCON) used an injection of dsRNA specific to a non-target viral gene. (**a**) Dose-dependent experiment on choice (%) between only dsPBAN-fed and dsPBAN-fed + PBAN-injected male and female insects using Y-tube olfactometer. PBAN was injected at doses of 1, 10, and 100 pmol/individual. In each treatment, three replications were used. In Y-tube olfactometry, the control chamber was kept vacant, and the treatment chamber contained dsRNA-treated male or female insects. Each replication used around 50 individual adults per treatment for Y-tube test. (**b**) NMB and LA concentrations were compared released by individual male and female *F. occidentalis* treated with dsPBAN + PBAN injection and only dsPBAN. Three replications were used per treatment. Different letters above standard deviation bars indicate significant differences among means at Type I error = 0.05 (LSD test). PBAN, pheromone biosynthesis activating neuropeptide, neryl (S)-2-methylbutanoate (NMB) and lavandulyl acetate (LA).

To test the effect of Fo-PBAN on the production of aggregation pheromone, its synthetic peptide was injected into male and female adults (Fig. 6). Test insects were treated with dsRNA specific to *Fo-PBAN*. Compared to the control (= dsCON), the RNAi-treated adults (‘0’ in PBAN addition) exhibited significant reductions in calling other adult thrips (Fig. 6a). The aggregation behavior was increased in a dose-dependent manner by the addition of the synthetic Fo-PBAN. To confirm the effect of Fo-PBAN on induction of aggregation behavior via the production of aggregation pheromone, the RNAi-treated adult thrips were injected with the highest amount (= 100 pmol/individual) of the synthetic peptide (Fig. 6b). The reduced amount of aggregation pheromone by the RNAi treatment was significantly rescued by the addition of the synthetic Fo-PBAN in both male and female adults.

### Prediction of biosynthetic pathway of aggregation pheromone in *F. occidentalis*

**Figure 7:**
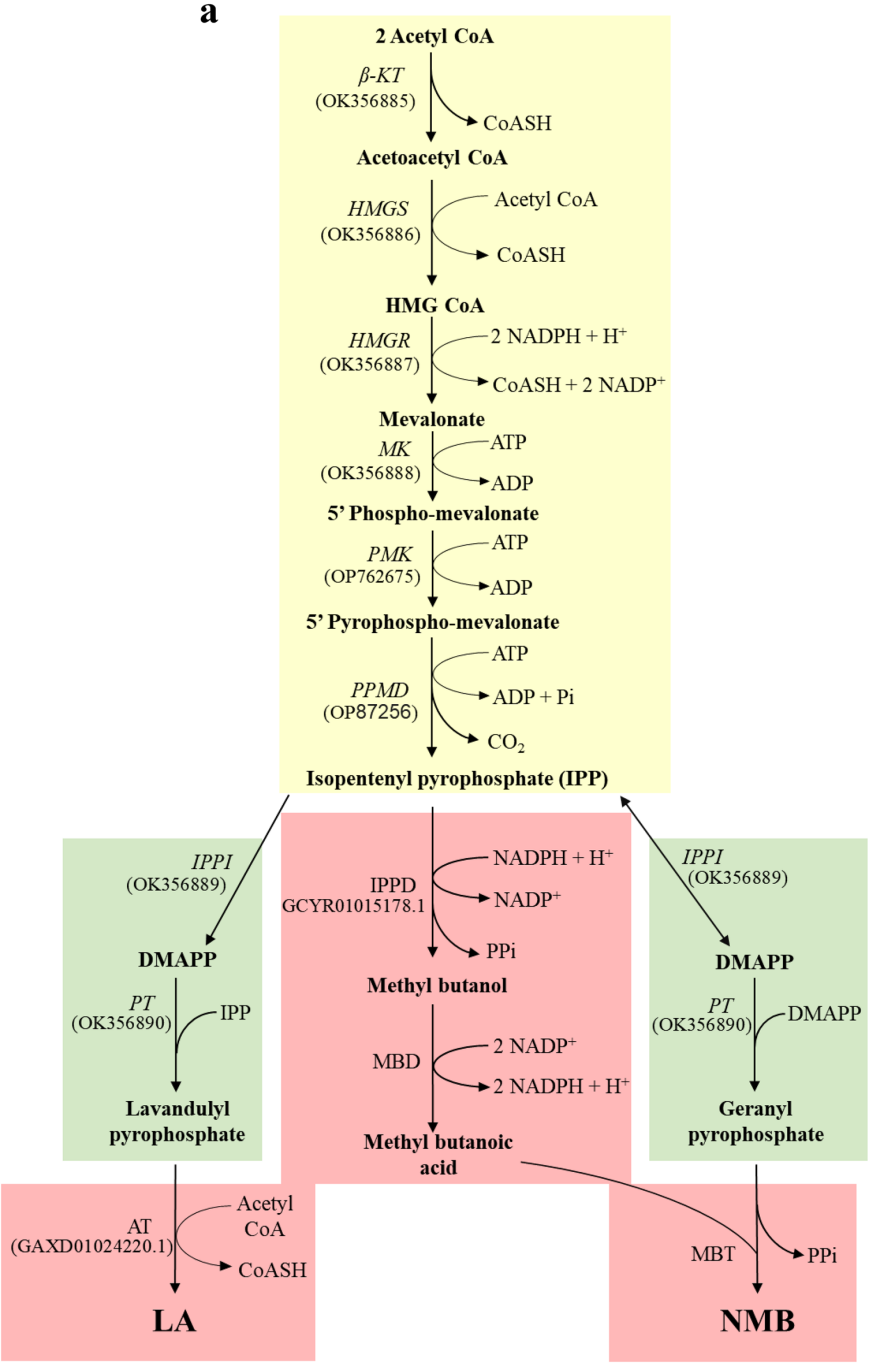

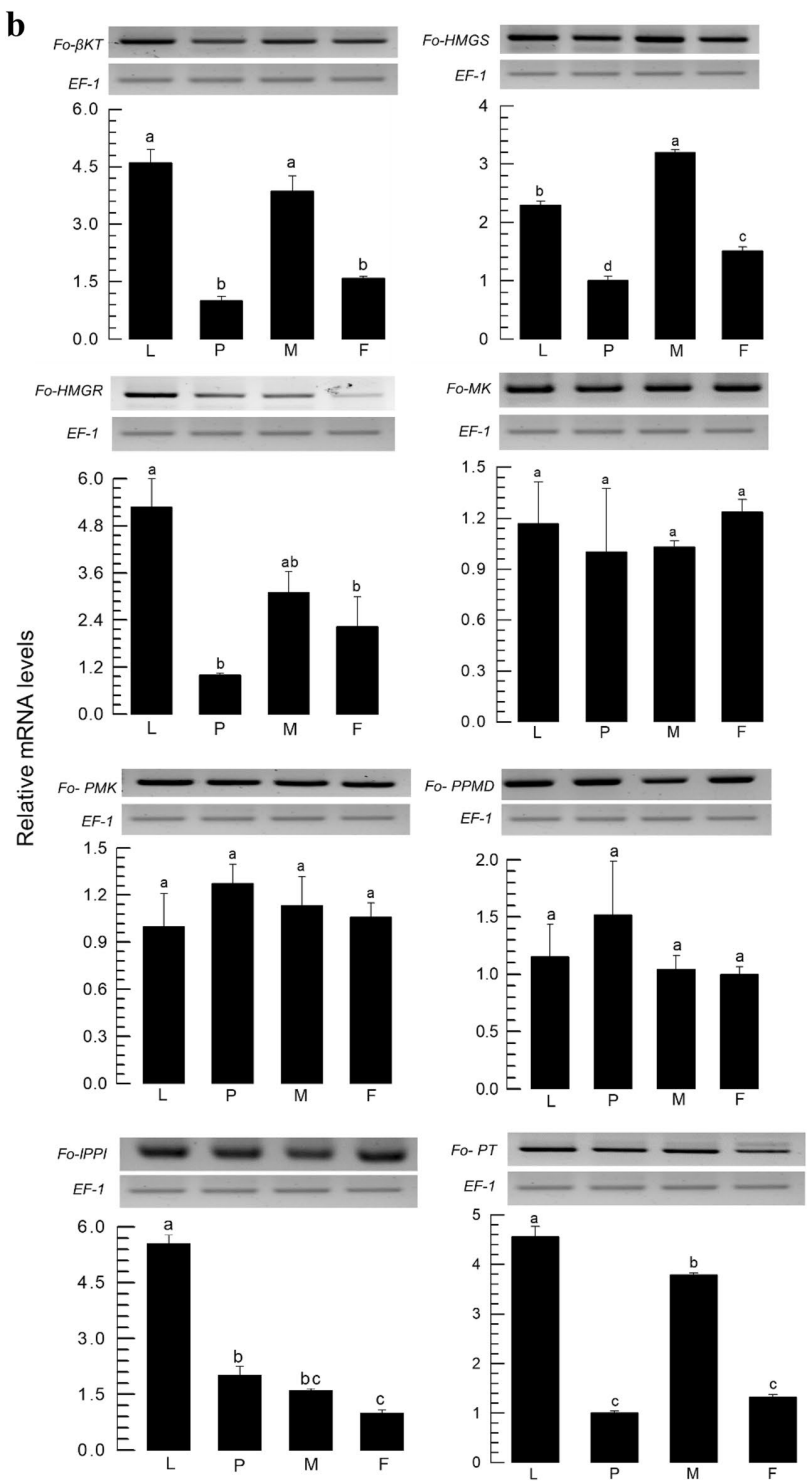
Prediction of biosynthesis pathway and mRNA expression analysis of aggregation pheromone. (**a**) A hypothetical biosynthetic pathways of neryl (*S*)-2-methylbutanoate (‘NMB’) and lavandulyl acetate (‘LA’), which are subdivided into three stages: stage 1 (yellow-colored) from acetyl-CoA to isopentenyl pyrophosphate (IPP), stage 2 (green-colored) from IPP to geranyl- or lavandulyl- pyrophosphate, and stage 3 (red-colored) from trans-acetylase or methylbutanoate to form LA or NMB, respectively. Figures in parentheses indicate GenBank accession numbers. The predicted genes include β-keto thiolase (KT), HMG CoA synthase (HMGS), HMG CoA reductase (HMGR), mevalonate kinase (MK), phosphomevalonate kinase (PMK), pyrophosphomevalonate decarboxylase (PPMD), isopentenyl pyrophosphate isomerase (IPPI), prenyl transferase (PT), isopentenyl pyrophosphate dehydrogenase (IPPD), methylbutanol dehydrogenase (MBD), methylbutanoic transferase (MBT), and acetyltransferase (AT). (**b**) Expression profiles of stage 1 and 2 genes in different developmental stages of *F*. *occidentalis*: larva (L), pupa (P), male adult (M), and female adult (F). An elongation factor, *EF1*, was used to normalize the expression level. Three replications were used per treatment. Different letters above or below standard deviation bars indicate significant differences among means at Type I error = 0.05 (LSD test). Gel images in full size are available in Figure S4.

Two components (NMB and LA) of the aggregation pheromone are terpenoids. Based on a terpenoid pheromone biosynthesis of coleopteran insects^15^, NMB and LA synthetic pathways were predicted using the corresponding orthologs (Table S1) encoded in *F. occidentalis* genome (Fig. 7a). This pathway consists of three stages: (1) acetyl CoA to isopentenyl pyrophosphate (IPP), (2) IPP to geranyl or lavandulyl pyrophosphate, and (3) transfer of methylbutanoate to NMB or transfer of acetylation to LA. Aside from the final stage, the identities of eight genes of all enzymes mediating the first two stages were confirmed by assessing their functional domains and phylogenetic analysis with other orthologs (Fig. S2). The eight genes associated were expressed in different developmental stages including male and female adults (Fig. 7b). Moreover, both male (Fig. S3a) and female (Fig. S3b) adults expressed all these eight genes with significant fluctuations in different ages.

### Inhibitory effects of individual RNAi treatments against the eight biosynthetic genes on aggregation pheromone biosynthesis in *F. occidentalis*

**Figure 8:**
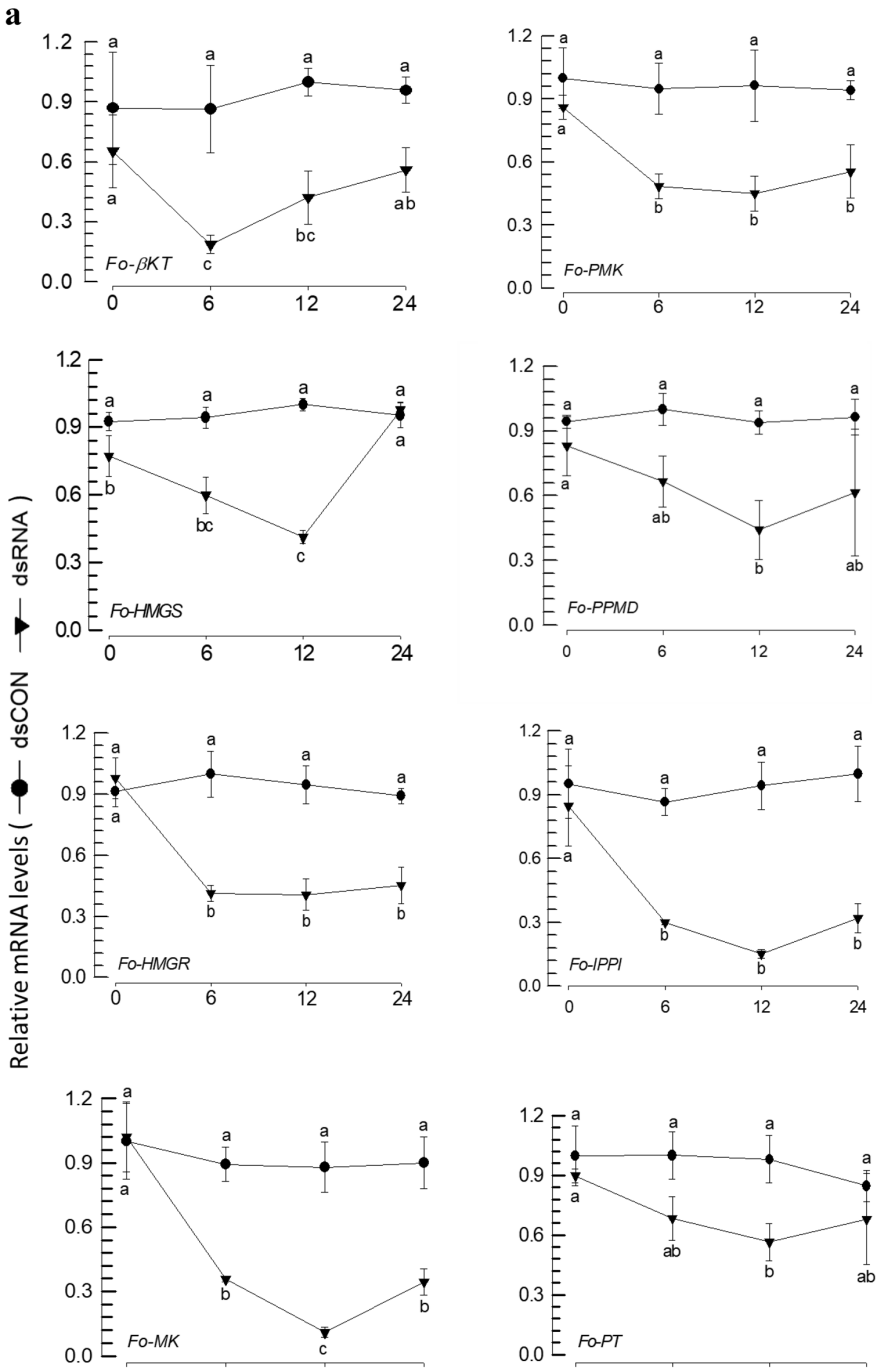

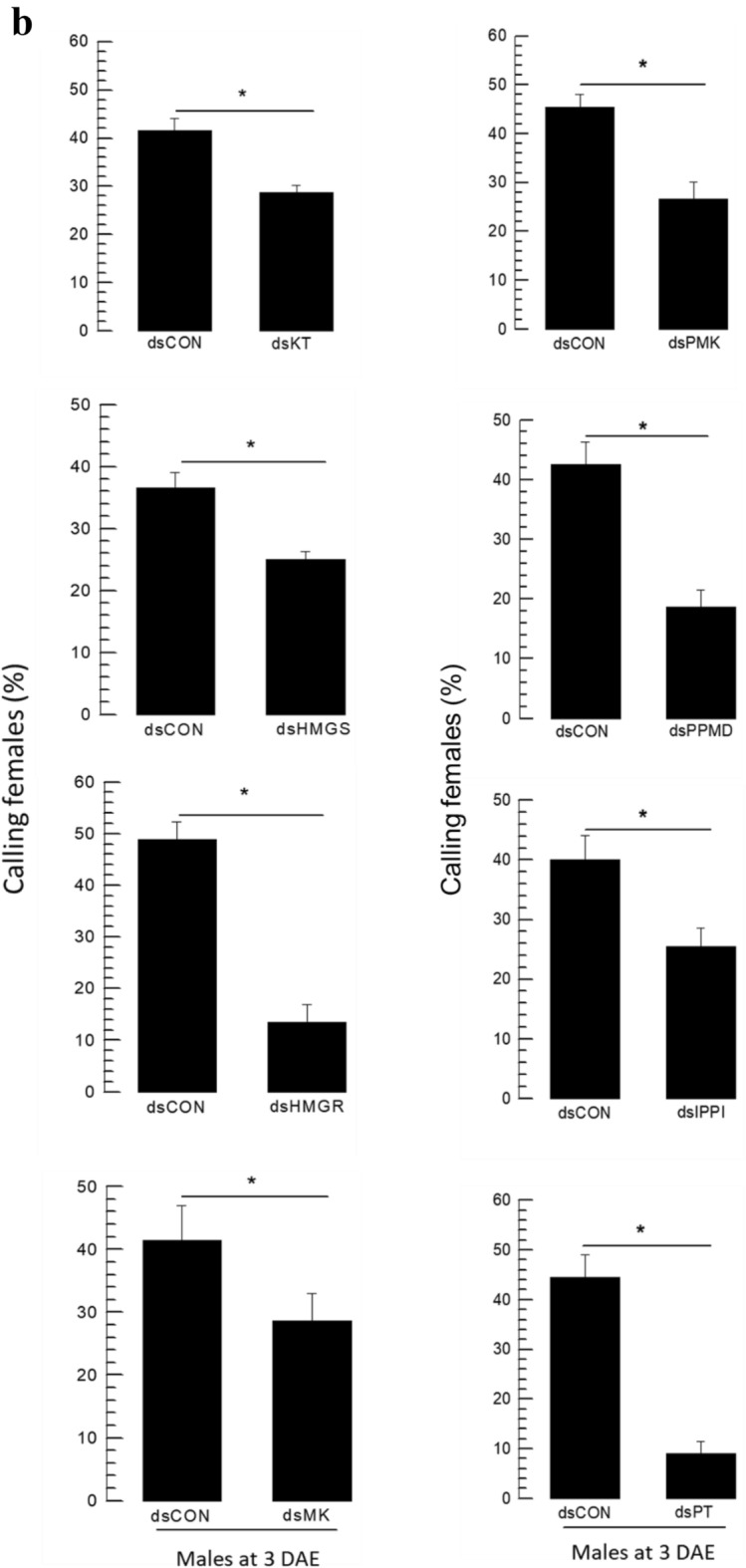

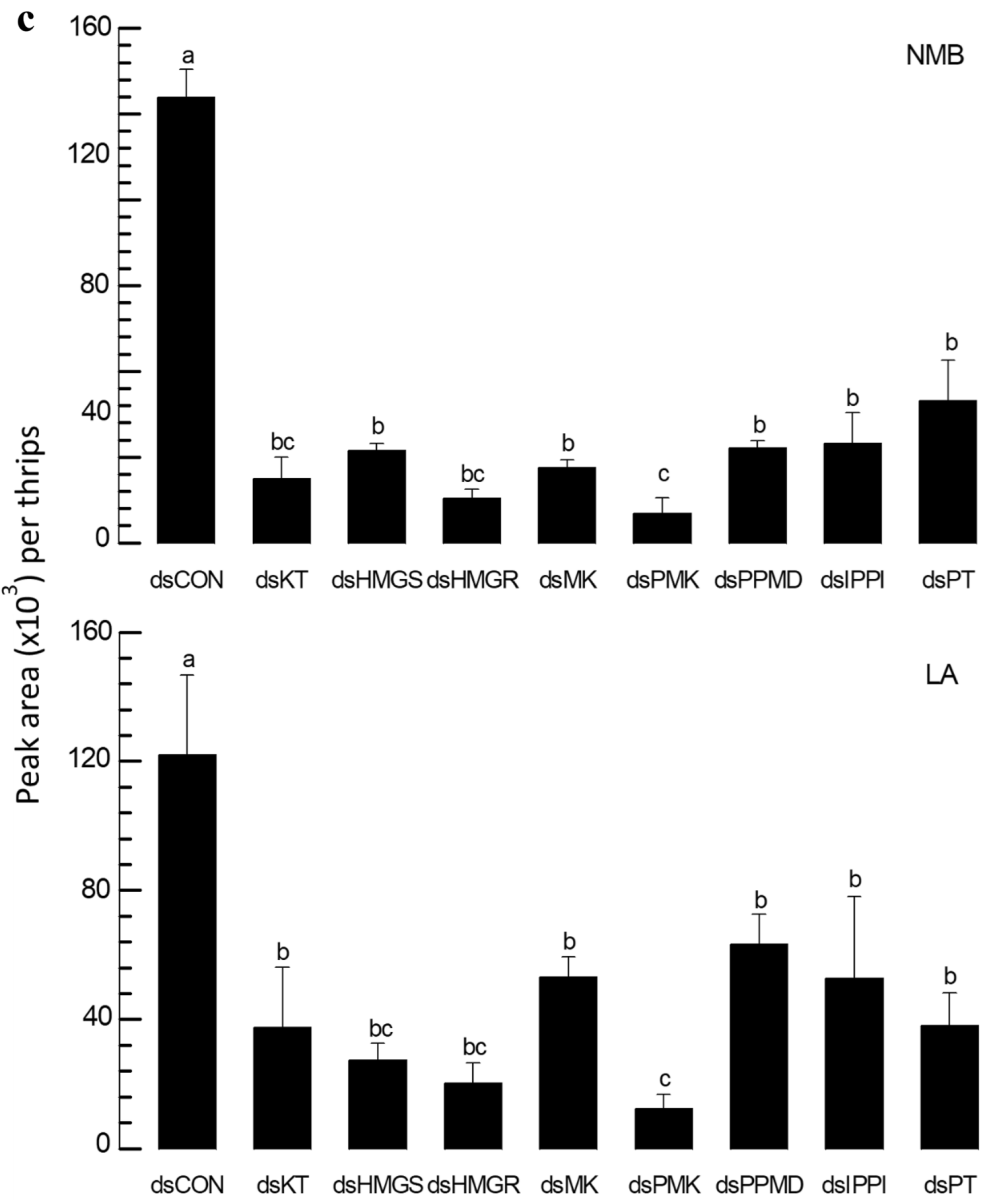
RNAi effect on eight predicted genes and neryl (S)-2-methylbutanoate (NMB) and lavandulyl acetate (LA) production of *F*. *occidentalis*. (**a**) Knockdown effect on eight genes after feeding of gene-specific dsRNA in male. A viral gene, *CpBV302*, was used as a control dsRNA (dsCON). An elongation factor, *EF1*, was used to normalize the expression level. In each treatment, three replications were used. (**b**) Behavior observation of the attraction of males to females after gene specific dsRNA application using a Y-tube olfactometer. In the olfactometer, the control chamber was kept vacant, and the treatment chamber contained dsRNA of feed male insect. Each replication used approximately 50 individual adults per treatment for Y-tube test. (**c**) Effect of gene specific dsRNA application on NMB and LA production in male *F. occidentalis*. All predicted genes had their expressions knocked down by RNAi using gene-specific dsRNA. RNAi was performed as feeding method. A viral gene, *CpBV302*, was used as a control dsRNA (dsCON). NMB and LA levels were measured using GC–MS. About 50 individual male thrips were transferred to a 10 mL amber vial. Inside the vial, two PDMS fiber tubes were kept for collecting volatile compounds. Each gene was considered as a treatment and each treatment was replicated three times. Different letters above standard deviation bars indicate significant differences among means at Type I error = 0.05 (LSD test). Keto thiolase (KT), HMG CoA synthase (HMGS), HMG CoA reductase (HMGR), mevalonate kinase (MK), phosphomevalonate kinase (PMK), pyro phosphomevalonate decarboxylase (PPMD), isopentenyl pyrophosphate delta-isomerase (IPPI), and prenyl transferase (PT).

The putative eight genes associated with aggregation pheromone biosynthesis were individually suppressed in their expressions by respective feeding gene-specific dsRNAs (Fig. 8). After 12-h feeding dsRNAs, the specific targets exhibited reductions in their expression levels of 40% (against *Fo-PT*) ~ 90% (against *Fo-MK*) compared to the dsCON-treated insects (Fig. 8a). Under the RNAi conditions, the treated males showed statistically significant (*P* < 0.05) impairments in calling females in all eight target genes (Fig. 8b). Furthermore, the RNAi treatments significantly (*P* < 0.05) prevented the production of aggregation pheromone in both components (Fig. 8c).

### PBAN induces expressions of the biosynthetic genes associated with aggregation pheromone in *F. occidentalis*

**Figure 9:**
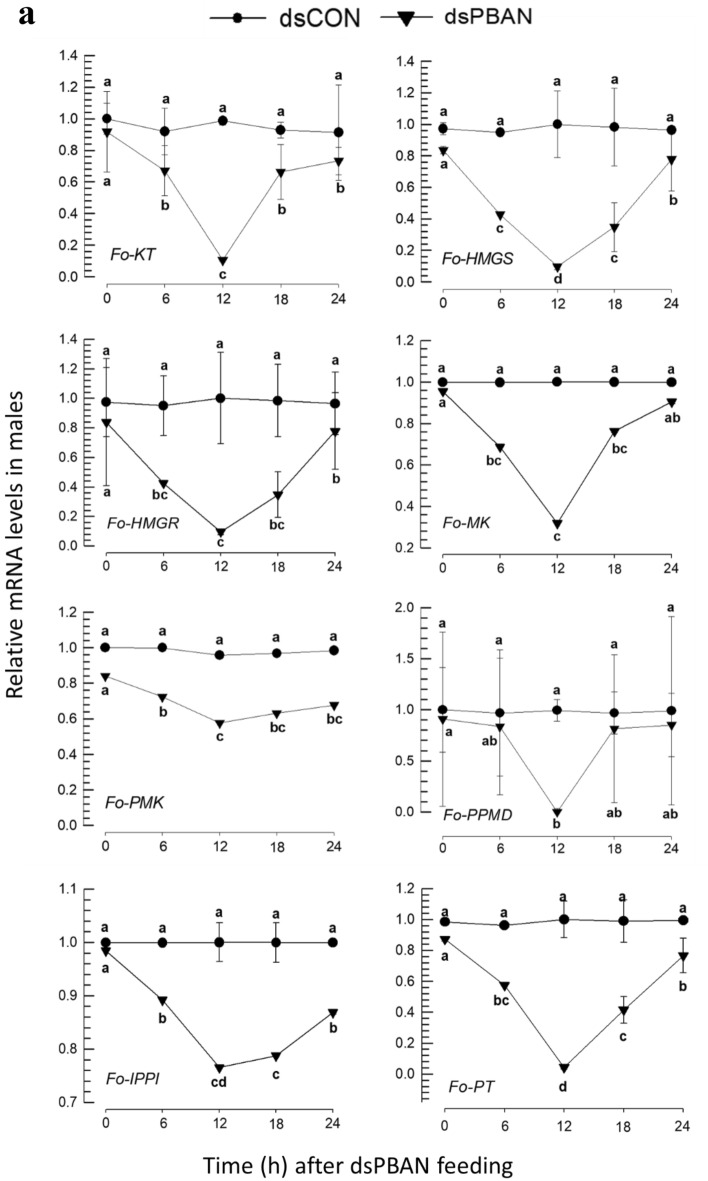

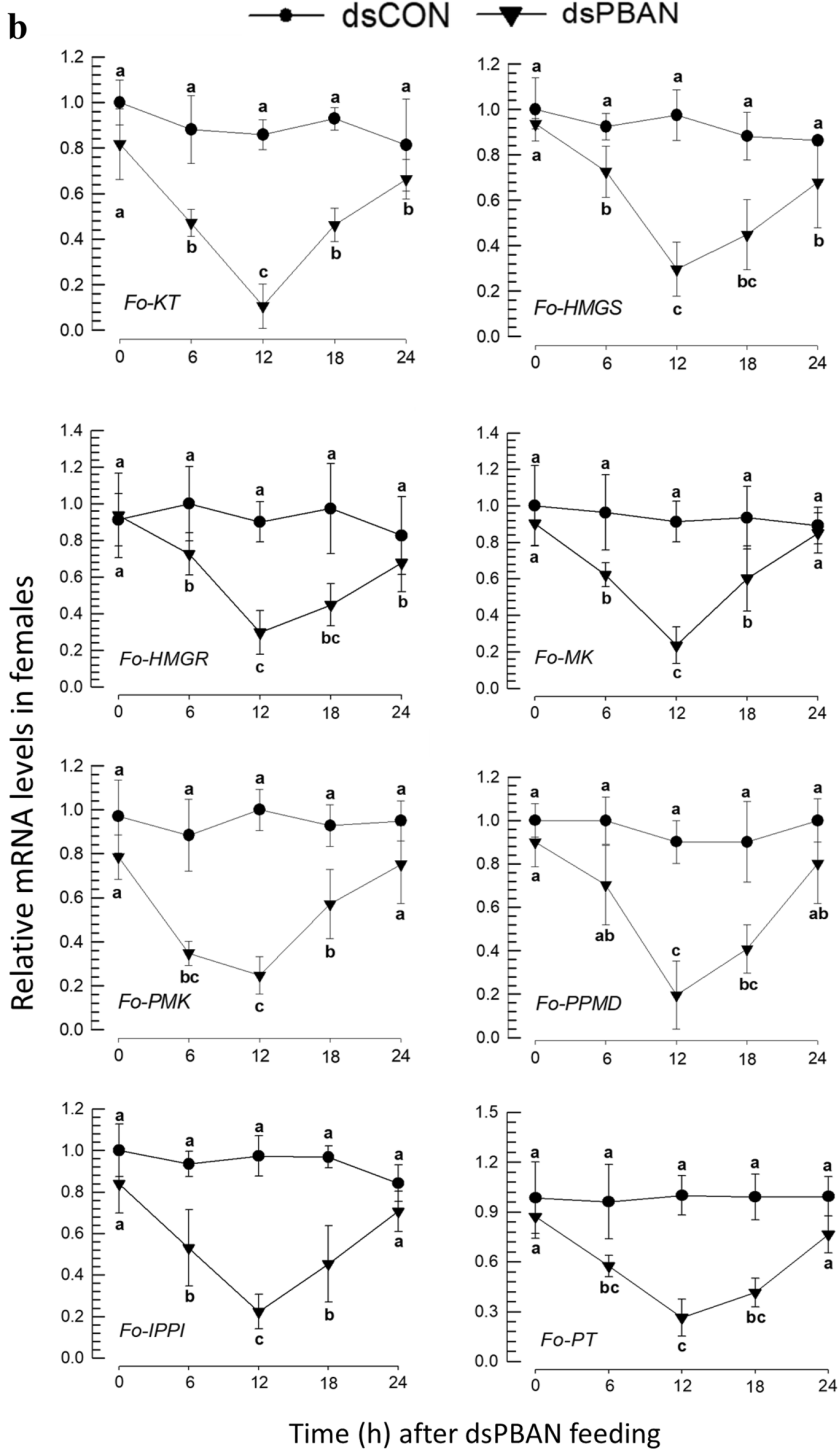
Knockdown effect of Fo-PBAN gene by RNAi on different genes related to aggregation pheromone biosynthesis. The knockdown effect was analyzed on (**a**) male and (**b**) female *F. occidentalis*. A viral gene, *CpBV302*, was used as a control dsRNA (dsCON). An elongation factor, *EF1*, was used to normalize the expression level. Three replications were used per treatment. Different letters above standard deviation bars indicate significant differences among means at Type I error = 0.05 (LSD test). Keto thiolase (KT), HMG CoA synthase (HMGS), HMG CoA reductase (HMGR), mevalonate kinase (MK), phosphomevalonate kinase (PMK), pyro phosphomevalonate decarboxylase (PPMD), isopentenyl pyrophosphate delta-isomerase (IPPI), and prenyl transferase (PT).

The influence of Fo-PBAN on the biosynthetic genes was assessed by analyzing changes in their expression levels (Fig. 9). The expression of *Fo-PBAN* was suppressed by feeding its specific dsRNA to males (Fig. 9a) and females (Fig. 9b). The RNAi treatment significantly (*P* < 0.05) reduced the expression levels of all eight target genes compared to those of the dsCON-treated insects.

## Discussion

Aggregation pheromone is used by insects to seek out food, protect from predators, and mate with the opposite sex^16^. Terry and Gardner^17^ observed that males of *F*. *occidentalis* form lek-like male-male aggressive aggregation behavior, wherein new females arrive and find mating partners from the aggregated males. This suggests that males and females are likely to use the aggregation pheromone for mating in *F. occidentalis*. Since the first identification of aggegation pheromone composition in *F. occidentlais*, similar components have been identified in related thrips^10,11^. These suggest that the biosynthetic machniery of the aggregation pheromones may be conserved among the thrips. However, little remains known about the biosynthetic pathways. This study used a genome of *F. occidentalis* to predict the biosynthetic machinery. Subsequent functional assays using RNAi supported the predicted biosynthetic pathway in *F. occidentalis*.

Aggregation pheromone has been known to be released only by male adults in *F. occidentalis*^9^. However, our current study surprisingly detected the aggregation pheromone in both females and males of *F. occidentalis*. To elaborate, this study used male and female adults at active age (= 3 days old after adult emergence) in mating behavior. In our assay, both male and female adults were shown to be attractive to other adults from Y-tube olfactometer behavior assays. The calling behavior was age-dependent, in which young adults at 3 days old after adult emergence were most active in both male and female adults. Subsequent analysis of mating behavior showed an age-dependent trend, suggesting that the calling signal of adults is linked with mating behavior of *F. occidentalis*. The actual amounts and relative mixture ratios of two pheromone components per individual did not differ much between male and female adults. This is clearly contradictory to the known facts in aggregation pheromone release from other related thrips. In *Thrips palmi*, a similar Y-tube olfactometer showed that males, but not females, send calling signal, and that subsequent analysis of headspace volatiles of only males contained the aggregation pheromone, (R)-lavandulyl 3-methyl-3-butenoate^11^. Another thrips, *Megalurothrips usitatu*, also supported the male-biased production of a single component aggregation pheromone, (2*E*,6*E*)-farnesyl acetate^13^. To justify our finding on aggregation pheromone synthesis and release in both sexes, this study further analyzed the biosynthetic machinery of *F. occidentalis*.

PBAN is a neuropeptide used to trigger pheromone biosynthesis in insects^18^. A PBAN gene (*Fo-PBAN*) was identified from *F. occidentalis* genome. Its transcript contains three neuropeptides including PBAN. The minimal sequence required to stimulate pheromone biosynthesis was the five amino acid with a C-terminal amidated end, which is the active core of the pyrokinin peptide family^19^. The C-terminal Fo-PBAN contains the conserved sequence, FSPRL. It is expressed in all developmental stages, including both male and female adults. Both male and female adults expressed the PBAN gene in a diel rhythm at daytime. Visual cues in addition to olfactory signals represent a major sensing system for locating hosts in *F. occidentalis*, wherein the visual recognition includes specific color and shape of the objects^20^. Using an LED light source, its color discrimination was further analyzed, and its color detection of three types of photoreceptors specific to blue, green, and UV was suggested^21^. This indicates that *F. occidentalis* exhibits host-searching behavior at daytime. Indeed, Kim^22^ showed that most matings of *F. occidentalis* occur in daytime under the control of circadian clock genes. These suggest that PBAN mediates the mating behavior of *F. occidentalis* by stimulating aggregation pheromone synthesis and release.

PBAN played a crucial role in triggering the aggregation pheromone biosynthesis in both male and female adults of *F. occidentalis*. RNAi of *Fo-PBAN* expression impaired calling behavior of young adults in both sexes by suppressing aggregation pheromone biosynthesis. In lepidopteran insects, PBAN is well known to stimulate sex pheormone production^23^. It is a neuropeptide that is produced in the subesophageal ganlion and belongs to a diverse group of peptides found across all insect orders^24^. These neuropeptides include pyrokinins mediating muscle contraction^25^ and diapause hormone inducing embryonic diapause in *Bombyx mori*^26^. In Heliothine moths, the diapause hormone plays a role in breaking pupal diapause^27^. The other functions of the peptides include acceleration of puparium formation in some higher Diptera^28^ and cuticle melanization in moth larvae^29,30^. Although PBAN mediates the production of sex pheromone in lepidopteran moths, it is unclear whether it plays a mediating role in other pheromone biosynthesis. Choi and Vander Meer^31^ report that the trail pheromone of the fire ant, *Solenopsis invicta*, is produced in Dufour's gland under the control of PBAN. RNAi knockdown of PBAN gene (in subesophageal ganglia) or PBAN receptor gene expression in the gland has been shown to inhibit trail pheromone biosynthesis. Our current study extends the physiological roles of PBAN by stimulating aggregation pheromone production in *F. occidentalis*.

This study proposed a biosynthetic pathway of the two components of *F. occidentalis* aggregation pheromone based on the fact that the components are terpenoids. The pathway was separated into three stages, in which the first stage from acetyl CoA to isopentenyl pyrophosphate (IPP) or dimethylallyl pyrophosphate (DMAPP) is well established in the aggregation pheromone production of other insects^32^. Subsequent combinatorial condensation of IPP or DMAPP leads to various terpenoids. Our current study analyzed the putative orthologs of *F. occidentalis* mediating the first and second biosynthetic stages of the aggregation pheromone and showed that their expressions were required for the pheromone production by individual RNAi treatments. However, the third stage enzymes mediating acetylation for LA production or transfer of methyl butanoate for NMB were not assessed because of the lack of corresponding orthologs. These transferases need to be clarified in future research.

RNAi of *Fo-PBAN* expression suppressed the expressions of the biosynthetic machinery genes at least for a day after the dsRNA treatment in *F. occidentalis*. It led to significant reduction in aggregation behavior of the treated adults. In moths, PBAN activates pheromone gland through a specific PBAN receptor, which is a membrane protein containing 7-transmembrane domain^33^. The functional binding of PBAN to its receptor elicits the intracellular signaling pathway via Ca^2+^ or cAMP to activate the biosynthetic machinery^23^. In addition, RNAi of *PBAN* expression led to reduced transcript levels associated with sex pheromone biosynthesis in *Spodoptera liture*^34^. Similarly, RNAi of PBAN receptor gene led to a significant reduction in fecundity, which presumably suppressed the pheromone biosynthetic machinery in *S. frugiperda*^35^. These sugggest that Fo-PBAN mediates the biosynthetic machinery by activating biosynthetic enzymes and up-regulating their gene expressions in the production of the aggregation pheromone.

Altogether, our current study suggests a novel function of PBAN in activating aggregation pheromone production by up-regulating its biosynthetic gene expressions. Moreover, female adults as well as males produce the aggregation pheromone in *F. occidentalis*, and they exhibit similar attractiveness to call adults for aggregation. This study reports a novel physiological function of PBAN in stimulating aggregation pheromone biosynthesis.

## Experimental procedures

### Insect rearing

A laboratory colony of *F. occidentalis* was obtained as a donation from the National Academy of Agricultural Sciences (Jeonju, Korea), and this was confirmed to be classified into G strain of the thrips^3^. Its rearing proceeded according to the method described in a previous study^3^. Our standard conditions in the rearing room are 25 ± 2 °C, 16L:8D photoperiod and 65 ± 5% relative humidity. A circular breeding container (100 mm × 40 mm, SPL, Korea) was used to rear thrips from eggs to adults.

### Chemicals

Two aggregation pheromone components (NMB and LA) were synthesized and purchased from AD, Inc. (Andong, Korea). Each compound was a racemic mixture and of > 99% purity^4^. PBAN of *F. occidentalis* (> 95% purity) was synthesized by Peptides 2.0 (Chantilly, VA, USA). Phosphate-buffered saline (PBS) was prepared with 100 mM phosphate containing 0.7% NaCl and adjusted to pH 7.4.

### Bioinformatics

All DNA sequences were obtained from GenBank—with the accession numbers listed in Table S2—and analyzed using BlastN tool (http://blast.ncbi.nlm.nih.gov/Blast.cgi). To get the open reading frame (ORF) of the collected sequences, the ORF finder tool from NCBI was used (https://www.ncbi.nlm.nih.gov/orffinder/). Prediction of the protein domain structure was done using EMBL-EBI (www.ebi.ac.uk) and Pfam (http://pfam.xfam.org). Phylogenetic analysis was performed using the Neighbor-Joining method and the Poisson correction model using MEGA6.06 software (www.megasoftware.net). Bootstrapping values were obtained with 1,000 replications to test the supports on each node in the resulting phylogenetic tree. For sequence alignment, the ClustalW program of MegAlign (DNASTAR, Version 6.0, Madison, WI, USA) was used.

### RNA extraction, cDNA synthesis, and PCRs

Total RNA was extracted from whole bodies of *F. occidentalis* in different developmental stages—i.e., ~ 50 larvae, ~ 30 pupae, and ~ 25 adults—using Trizol reagent (Invitrogen, Carlsbad, CA, USA). In diel rhythm analysis, insect samples were collected from the rearing colonies at every 3 h after light-on under the 16 h photoperiod. cDNA preparation and subsequent PCR followed the method described in a previous study^36^ using specific primers (Table S3).

### RNA interference (RNAi)

Double-stranded RNAs (dsRNAs) were used for RNAi. To prepare dsRNAs specific to different genes, template DNAs were amplified with forward and reverse gene-specific primers containing T7 promoter sequence at their 5ʹ ends. The resulting T7 promoter-tagged template DNAs were used to construct dsRNAs using MEGAscript RNAi kit (Ambion, Austin, TX, USA). The newly formed dsRNAs were mixed with Metafectene PRO (Biontex, Plannegg, Germany), a transfection reagent, at a 1:1 ratio, and incubated at 25 °C for 30 min to form liposomes. These dsRNAs were treated by the feeding delivery method. The bean diet was soaked in dsRNA suspension for 20 min. After removing excess moisture, the treated beans were supplied to 10 individuals in a breeding dish (= an experimental unit). Each treatment was replicated three times. A viral gene, *CpBV302*^37^, was used to prepare control dsRNA (= dsCON). After 24 h feeding, untreated fresh beans were supplied to the test thrips. RNAi efficiencies were measured for 24 h after dsRNA feeding by RT-qPCR.

### Mating behavior assay

Before measuring the mating behavior, pupae were separated from the colony and transferred to a 1.5 mL microtube (Axygen, Union City, CA, USA). Newly emerged adults were separated from the rearing colony and age-graded by days after emergence. The adults were individually provided with a diet bean (0.2 × 0.2 cm) until being used for assay. A mating arena used a cap of 1.5 mL microtube covered with cover glass (18 × 18 mm, Marienfeld, Königshofen, Germany) and included a pair of adults under a stereomicroscope for observation. Each mating behavior was examined for 10 min after the introduction of both males and females under the conditions of 25 ± 1 °C temperature and 60 ± 5% relative humidity under a dark condition with an infrared light (Infrared light 150W, Couyor, China) illumination. Copulation behavior within 10 min in the arena was recorded as a successful mating. Each observation was replicated 10 times with every fresh adult.

### Y-tube olfactometer assay

For the behavioral choice test of *F. occidentalis*, a Y-tube olfactometer was used. The transparent Y-tube glass consisted of a 100 mm stem with two 80 mm arms separated by a 45° angle. The internal diameter of the Y-tube was 10 mm. Humidified air filtered through activated charcoal was supplied into the two arms. All behavioral assays were performed in a dark room under an infrared light (1000 lx illumination) at 25 ± 1 °C and 65% relative humidity. In each run, 50 adults were applied at the bottom of the stem tube. Test insects or compounds were installed at one side of the arm tube. The running time of each run was 10 min. Positive insects were judged as those passing over 60 mm from the branching site. Each treatment consisted of four replications while changing the installation position of the test samples between two arms in each replication.

### PBAN injection of male and female thrips

PBAN was dissolved in PBS. Male and female adults (3 days after emergence) were fed with dsRNA in the manner described above. At 12 h after the RNAi treatment, PBAN was injected in a volume of 50 nL to each of the individual treated thrips using a nanoinjector (World Precision Instrument, Sarasota, FL, USA). The micro-injection proceeded according to the method described by Raffin et al.^38^ with slight modification. Briefly, during injection, test thrips was immobilized on two-sided sticky tape. A microcapillary tip was inserted through the membranous integument between the thorax and the abdomen. Injected thrips were then detached from the tape and provided with diet. After 6 h of incubation at room temperature, the treated insects were then used for behavioral or chemical analysis.

### Analysis of volatile compounds using thermal desorption (TD)-coupled with gas chromatography-mass spectrometry (GC–MS)

Headspace volatiles of the test thrips were trapped in polydimethylsiloxane (PDMS) tubing and analyzed according to a previously described method^39^. Briefly, PDMS tube pieces (1 cm in length) were placed directly over the adult thrips (*n* = 50) in a 10 mL amber vial (22.5 × 46 mm) (Sci Lab, Seoul, Korea) for 5 h at room temperature. An individual piece of PDMS tubing was placed in an 89 mm glass TD tube, desorbed under a stream N_2_ gas at 230 °C for 8 min (TD-30, Shimadzu, Kyoto, Japan), then directly subjected to GC–MS (GCMS-QP2020, Shimadzu, Kyoto, Japan). Volatiles were separated with a Rtx-5MS capillary column (30 m × 0.25 mm, 0.25 μm film thickness: Shimadzu, Kyoto, Japan) on split mode at a 1/20 ratio under the constant linear velocity of 40 cm s^−1^ with helium as a carrier gas. The GC oven began at 40 °C for 5 min, increased to 185 °C at a rate of 5 °C/min, and then increased again to 280 °C at a rate of 30 °C/min; the oven was kept at 280 °C for 0.83 min. Electron impact (an ionization energy of 70 eV) spectra were conducted in scan mode from *m/z* 33 to 400. Identification of NMB and LA were confirmed by matching mass spectra from the NIST 14 library and authenticated standards.

### Statistical analysis

All data for continuous variables were subjected to one-way analysis of variance (ANOVA) using PROG GLM in the SAS program^40^. Percent data were subjected to arcsine transformation and used for ANOVA. Means were compared with the least significant difference (LSD).

## Supplementary Information

Supplementary Information.

## Data Availability

All data generated or analysed during this study are included in this published article and its supplementary information file. The genome sequence datasets generated and/or analysed during the current study are available in GenBank repository using accession numbers in Table S2.
