# Supplementary material for: A novel physiological function of pheromone biosynthesis-activating neuropeptide in production of aggregation pheromone
Source: Sci Rep. 2023 Apr 5;13:5551. doi: 10.1038/s41598-023-32833-9 (PMC10076286; doi:10.1038/s41598-023-32833-9)
Supplement: Supplementary file 1 — Supplementary Information. [file 41598_2023_32833_MOESM1_ESM.docx]

**Supplementary Information**

**Table S1.** Primers used in this study

**Table S2.** GenBank accession numbers of PBANs found in different insects

**Table S3.** Prediction of aggregation pheromone biosynthetic pathway components in *F. occidentalis*

**Figure S1.** Sequence alignment of PBANs in different insects. GenBank accession numbers and species acronyms are described in Table S3.

**Figure S2.** Different genes associated with aggregation pheromone biosynthesis pathway: **a** Prediction of the functional domain of eight genes. **b** Phylogenetic relationship of eight genes with other insect species sequence of different orders. The tree was constructed with the Neighbor-Joining method using MEGA6.06. Bootstrapping values on branches were obtained with 1,000 replications.

**Figure S3.** Expression profiles of eight genes associated with aggregation biosynthesis in male **a** and female **b** *F. occidentalis* (Fo) for 14 days after adult emergence: β-keto thiolase (KT), HMG CoA synthase (HMGS), HMG CoA reductase (HMGR), mevalonate kinase (MK), phosphomevalonate kinase (PMK), pyrophosphomevalonate decarboxylase (PPMD), isopentenyl pyrophosphate isomerase (IPPI), and prenyl transferase (PT). An elongation factor, *EF1*, was used to normalize the expression level. Three replications were used per treatment. Different letters above standard deviation bars indicate significant differences among means at Type I error = 0.05 (LSD test).

**Figure S4**. Full size gel images in Figure 7b. β-Keto thiolase (βKT), HMG CoA synthase (HMGS), HMG CoA reductase (HMGR), mevalonate kinase (MK), phosphomevalonate kinase (PMK), pyro phosphomevalonate decarboxylase (PPMD), isopentenyl pyrophosphate delta-isomerase (IPPI), and prenyl transferase (PT) of *F. occidentalis* (Fo).

**Table S1.** Prediction of aggregation pheromone biosynthetic pathway components in *F. occidentalis*

| Annotation | Acronym | GenBank | Blast search | |
| --- | --- | --- | --- | --- |
|  |  |  | **Species** | **E-value** |
| β-keto-Thiolase | *Fo- KT* | GCYR01026358.1 | *T. palmi* | 0.0 |
| HMG-CoA synthase | *Fo-HMGS* | XM_026435472.1 | *T. palmi* | 0.0 |
| HMG-CoA reductase | *Fo-HMGR* | XM_026425781.1 | *T. palmi* | 0.0 |
| Mevalonate kinase | *Fo-MK* | XM_026423073.1 | *T. palmi* | 2e-72 |
| Phosphomevalonate kinase | *Fo-PMK* | XM_026428324.1 | *T. palmi* | 2e-59 |
| Pyrophosphomevalonate decarboxylase | *Fo-PPMD* | GCYR01018019.1 | *T. palmi* | 0.0 |
| Isopentenyl-diphosphate isomerase | *Fo-IPPI* | XM_026424735.1 | *T. palmi* | 2e-84 |
| Prenyl transferase | *Fo-PT* | XM_026424759.1 | *T. palmi* | 2e-129 |

**Table S2.** GenBank accession numbers of different PBANs

| Organism Name | GenBank accession number | Acronym | Order |
| --- | --- | --- | --- |
| *Frankliniella occidentalis* | OP787255 | *Fo-PBAN* | Thysanoptera |
| *Anopheles albimanus* | XP_035796452.1 | *Aab-PBAN* | Diptera |
| *Neodiprion lecontei* | XP_015522568.1 | *Nl-PBAN* | Diptera |
| *Culex quinquefasciatus* | XP_001847418.1 | *Cqq-PBAN* | Diptera |
| *Culex pipiens pallens* | XP_039452722.1 | *Cpp-PBAN* | Diptera |
| *Aedes aegypti* | XP_001662212.2 | *Aae-PBAN* | Diptera |
| *Anopheles merus* | XP_041762518.1 | *Am-PBAN* | Diptera |
| *Aedes albopictus* | XP_029733733.1 | *Aal-PBAN* | Diptera |
| *Anopheles arabiensis* | XP_040151912.1 | *Aar-PBAN* | Diptera |
| *Cephus cinctus* | XP_015600657.1 | *Cci-PBAN* | Diptera |
| *Solenopsis carolinensis* | ADI88480.1 | *Sc-PBAN* | Hymenoptera |
| *Monomorium pharaonis* | XP_012524735.1 | *Mp-PBAN* | Hymenoptera |
| *Ooceraea biroi* | XP_011330631.1 | *Ob-PBAN* | Hymenoptera |
| *Nomia melanderi* | XP_031838828.1 | *Nm-PBAN* | Hymenoptera |
| *Ceratina calcarata* | XP_017875879.1 | *Cc-PBAN* | Hymenoptera |
| *Frieseomelitta varia* | XP_043522355.1 | *Fv-PBAN* | Hymenoptera |
| *Apis florea* | XP_003693827.1 | *Af-PBAN* | Hymenoptera |
| *Apis cerana* | XP_028520746.1 | *Ac-PBAN* | Hymenoptera |
| *Apis laboriosa* | XP_043799836.1 | *Al-PBAN* | Hymenoptera |
| *Trichoplusia ni* | XP_026747553.1 | *Tn-PBAN* | Lepidoptera |
| *Pararge aegeria* | XP_039762919.1 | *Pa-PBAN* | Lepidoptera |
| *Carposina sasakii* | UCJ01501.1 | *Cs-PBAN* | Lepidoptera |
| *Maruca vitrata* | AFX71575.1 | *Mv-PBAN* | Lepidoptera |
| *Maniola jurtina* | XP_045783104.1 | *Mj-PBAN* | Lepidoptera |
| *Ostrinia furnacalis* | XP_028168133.1 | *Ofu-PBAN* | Lepidoptera |
| *Ostrinia nubilalis* | AOY34010.1 | *On-PBAN* | Lepidoptera |
| *Galleria mellonella* | XP_026759976.1 | *Gm-PBAN* | Lepidoptera |
| *Omphisa fuscidentalis* | AFP87384.1 | *Of-PBAN* | Lepidoptera |
| *Heliothis virescens* | AAO20095.1 | *Hv-PBAN* | Lepidoptera |
| *Spodoptera frugiperda* | XP_035457345.1 | *Sf-PBAN* | Lepidoptera |
| *Chlumetia transversa* | AIY72749.1 | *Ct-PBAN* | Lepidoptera |
| *Manduca sexta* | XP_030038326.2 | *Ms-PBAN* | Lepidoptera |
| *Spodoptera exigua* | AAT64424.1 | *Se-PBAN* | Lepidoptera |
| *Helicoverpa armigera* | AAL05596.1 | *Ha-PBAN* | Lepidoptera |
| *Samia ricini* | AAP41132.1 | *Sr-PBAN* | Lepidoptera |
| *Antheraea pernyi* | AAR17699.1 | *Ap-PBAN* | Lepidoptera |
| *Bombyx mandarina* | XP_028035013.1 | *Bma-PBAN* | Lepidoptera |
| *Aricia agestis* | XP_041984260.1 | *Aa-PBAN* | Lepidoptera |
| *Pieris brassicae* | XP_045526671.1 | *Pb-PBAN* | Lepidoptera |
| *Plutella xylostella* | AEP25400.1 | *Px-PBAN* | Lepidoptera |
| *Pieris rapae* | QDA95097.1 | *Pr-PBAN* | Lepidoptera |
| *Papilio machaon* | XP_045541162.1 | *Pp-PBAN* | Lepidoptera |
| *Bombyx mori* | AAB24327.1 | *Bm-PBAN* | Lepidoptera |
| *Tribolium castaneum* | XP_015835244.1 | *Tc-PBAN* | Coleoptera |
| *Tribolium madens* | XP_044257101.1 | *Tm-PBAN* | Coleoptera |
| *Aethina tumida* | XP_019871286.1 | *At-PBAN* | Coleoptera |
| *Dendroctonus ponderosae* | XP_019770936.1 | *Dp-PBAN* | Coleoptera |
| *Sitophilus oryzae* | XP_030756370.1 | *So-PBAN* | Coleoptera |

**Table S3.** Primers used in this study

| Genes | Orientation | Sequence (5’-3’) | Uses | Annealing temperature (^0^C) | Expected size (bp) |
| --- | --- | --- | --- | --- | --- |
| β-keto Thiolase | Forward | AACAGTCGCAGGGGAGATTG | RT-PCR  RT-qPCR | 52.0 | 209 |
|  | Reverse | GGGCTATGCCTTCCACTGTT |  |  |  |
| T7+ β-keto Thiolase | Forward | TAATACGACTCACTATAGGGAGAAACAGTCGCAGGGGAGATTG | RNAi | 52.0 | 255 |
|  | Reverse | TAATACGACTCACTATAGGGAGAGGGCTATGCCTTCCACTGTT |  |  |  |
| HMG CoA Synthase | Forward | GACAAGTCAACCATTGGGCG | RT-PCR  RT-qPCR | 52.0 | 236 |
|  | Reverse | GCAATGTCTCCTGCCACAAC |  |  |  |
| T7+ HMG CoA Synthase | Forward | TAATACGACTCACTATAGGGAGAGACAAGTCAACCATTGGGCG | RNAi | 52.0 | 282 |
|  | Reverse | TAATACGACTCACTATAGGGAGAGCAATGTCTCCTGCCACAAC |  |  |  |
| HMG CoA Reductase | Forward | CTAGCCACGACAGAGGGTTG | RT-PCR  RT-qPCR | 55.0 | 263 |
|  | Reverse | ATGTACAGTGAGCGTCCTGC |  |  |  |
| T7+ HMG CoA Reductase | Forward | TAATACGACTCACTATAGGGAGACTAGCCACGACAGAGGGTTG | RNAi | 55.0 | 309 |
|  | Reverse | TAATACGACTCACTATAGGGAGAATGTACAGTGAGCGTCCTGC |  |  |  |
| Mevalonate Kinase | Forward | ATGGCGGCCAGTCTTGATTT | RT-PCR  RT-qPCR | 52.0 | 260 |
|  | Reverse | AGGACTGTTCACATCCCACG |  |  |  |
| T7+ Mevalonate Kinase | Forward | TAATACGACTCACTATAGGGAGATGGAGACTCGTGGGATGTGA | RNAi | 52.0 | 306 |
|  | Reverse | TAATACGACTCACTATAGGGAGAAGGAATGGTTGCAGAGTGGGT |  |  |  |
| Phosphomevalonate Kinase | Forward | GCGTCTTTGTCGGACACTTTC | RT-PCR  RT-qPCR | 52.0 | 106 |
|  | Reverse | GAGCGACATCCGACGTAAAAC |  |  |  |
| T7+ Phosphomevalonate Kinase | Forward | TAATACGACTCACTATAGGGAGGCGTCTTTGTCGGACACTTTC | RNAi | 52.0 | 152 |
|  | Reverse | TAATACGACTCACTATAGGGAGGAGCGACATCCGACGTAAAAC |  |  |  |
| Pyrophosphomevalonate decarboxylase | Forward | GCTGTAGGCATGCAAAAGCA | RT-PCR  RT-qPCR | 52.0 | 125 |
|  | Reverse | AATGGAAGGCCACTTGCTGA |  |  |  |
| T7+ Pyrophosphomevalonate decarboxylase | Forward | TAATACGACTCACTATAGGGAGGCTGTAGGCATGCAAAAGCA | RNAi | 52.0 | 171 |
|  | Reverse | TAATACGACTCACTATAGGGAGAATGGAAGGCCACTTGCTGA |  |  |  |
| Isopentenyl-diphosphate Delta-isomerase | Forward | GGAATACGACACGCTGCTCA | RT-PCR  RT-qPCR | 52.0 | 126 |
|  | Reverse | AAATTTGCCATCACCGCTGTC |  |  |  |
| T7+ Isopentenyl-diphosphate Delta-isomerase | Forward | TAATACGACTCACTATAGGGAGAGGAATACGACACGCTGCTCA | RNAi | 52.0 | 172 |
|  | Reverse | TAATACGACTCACTATAGGGAGAAAAATTTGCCATCACCGCTGTC |  |  |  |
| Prenyl Transferase | Forward | CCAAGTGTGCTCTCACGTCT | RT-PCR  RT-qPCR | 52.0 | 268 |
|  | Reverse | TGAGTGAAACACGCAGCTCT |  |  |  |
| T7+ Prenyl Transferase | Forward | TAATACGACTCACTATAGGGAGACCAAGTGTGCTCTCACGTCT | RNAi | 52.0 | 314 |
|  | Reverse | TAATACGACTCACTATAGGGAGAATGAGTGAAACACGCAGCTCT |  |  |  |
| PBAN | Forward | CAACGACATCCCCTGGAAGC | RT-PCR  RT-qPCR | 52.0 | 118 |
|  | Reverse | GCCGTTAGCAATGTTGGACA |  |  |  |
| T7+ PBAN | Forward | TAATACGACTCACTATAGGGAGACAACGACATCCCCTGGAAGC | RNAi | 52.0 | 164 |
|  | Reverse | TAATACGACTCACTATAGGGAGAGCCGTTAGCAATGTTGGACA |  |  |  |
| Elongation Factor 1 | Forward | TCA AGG AAC TGC GTC GTG GAT | RT-qPCR | 52.0 | 160 |
|  | Reverse | ACA GGG GTG TAG CCG TTA GAG |  |  |  |

**
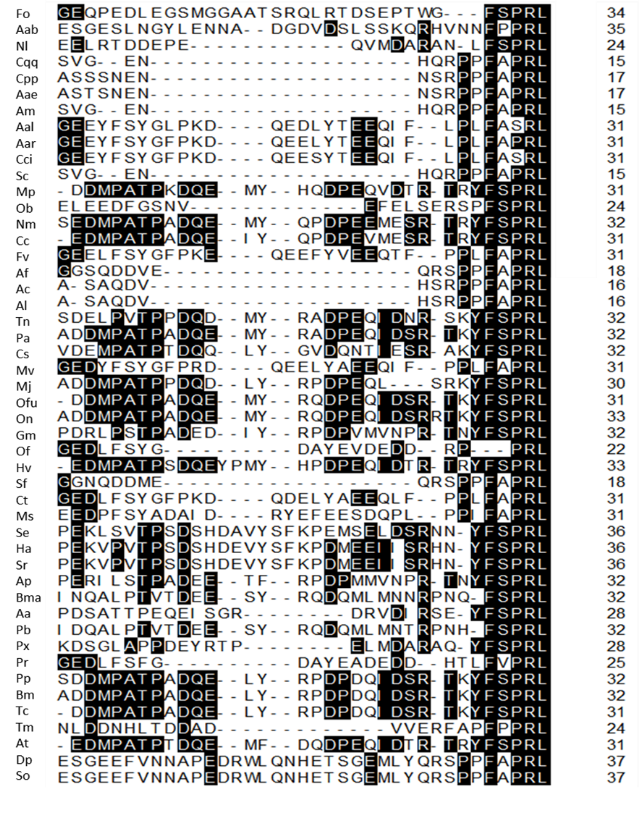
**

**Fig. S1**

**a**


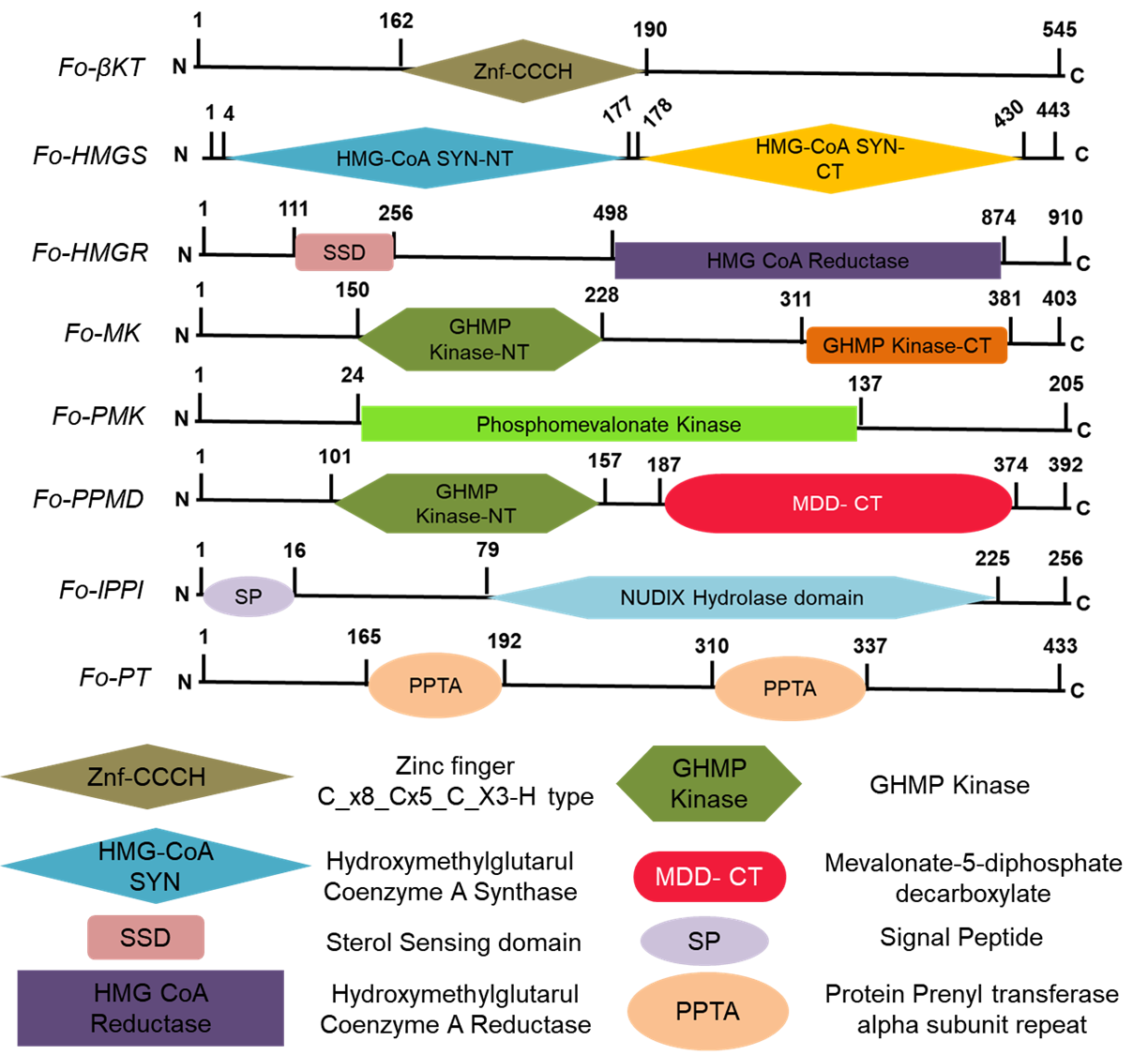


**b**


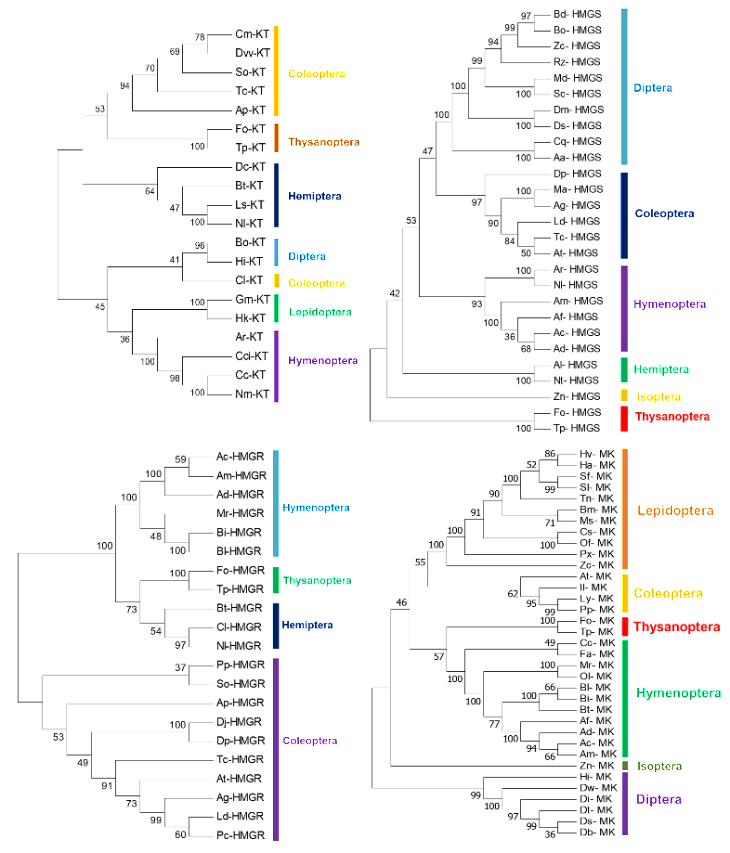


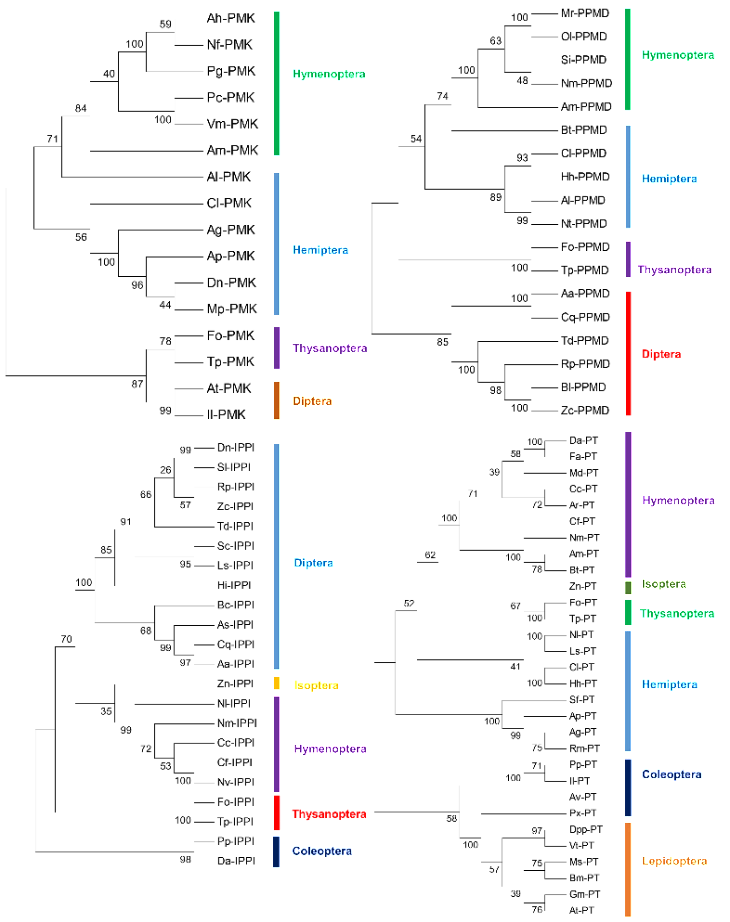


**Fig. S2**

**a**


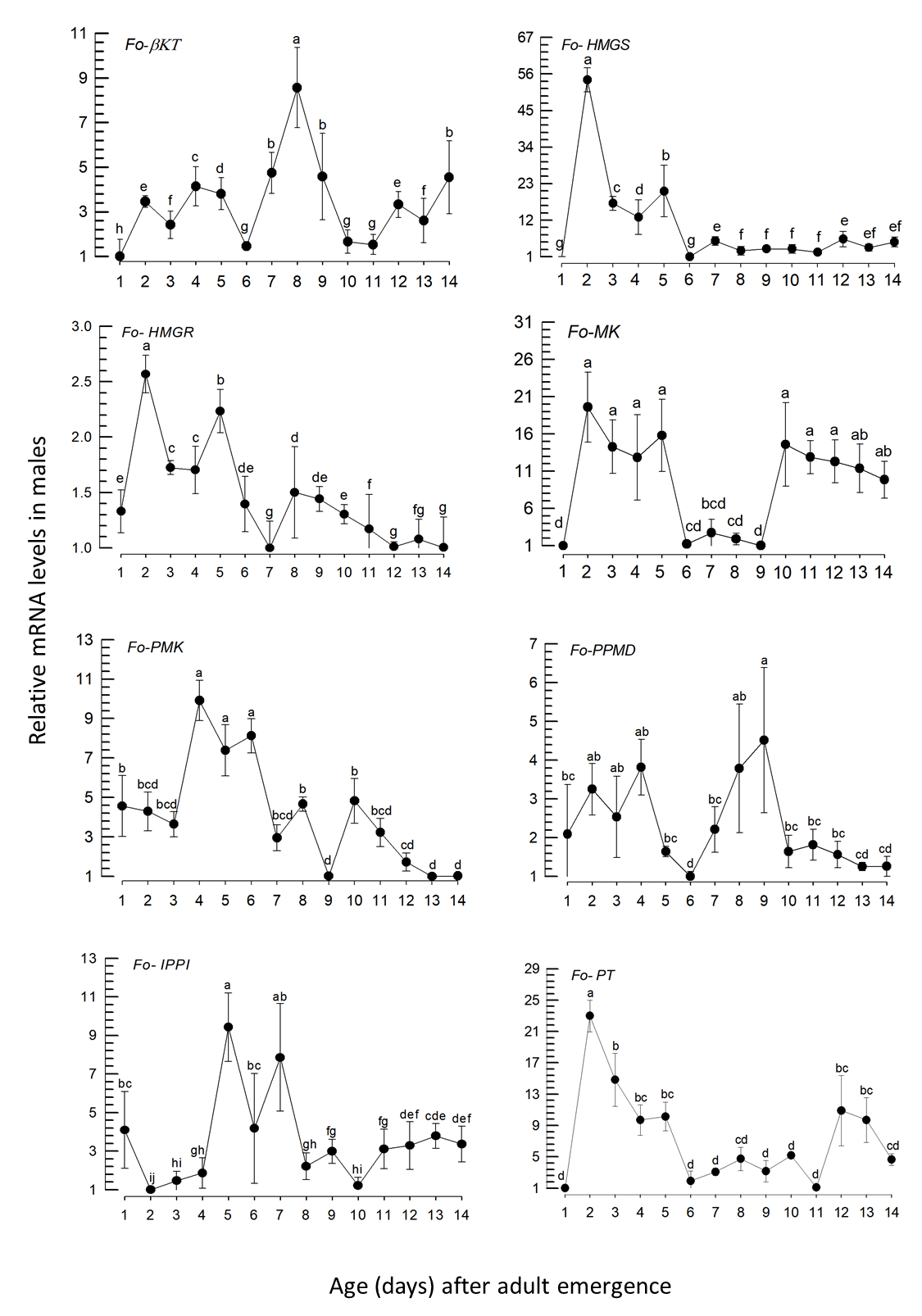


**b**


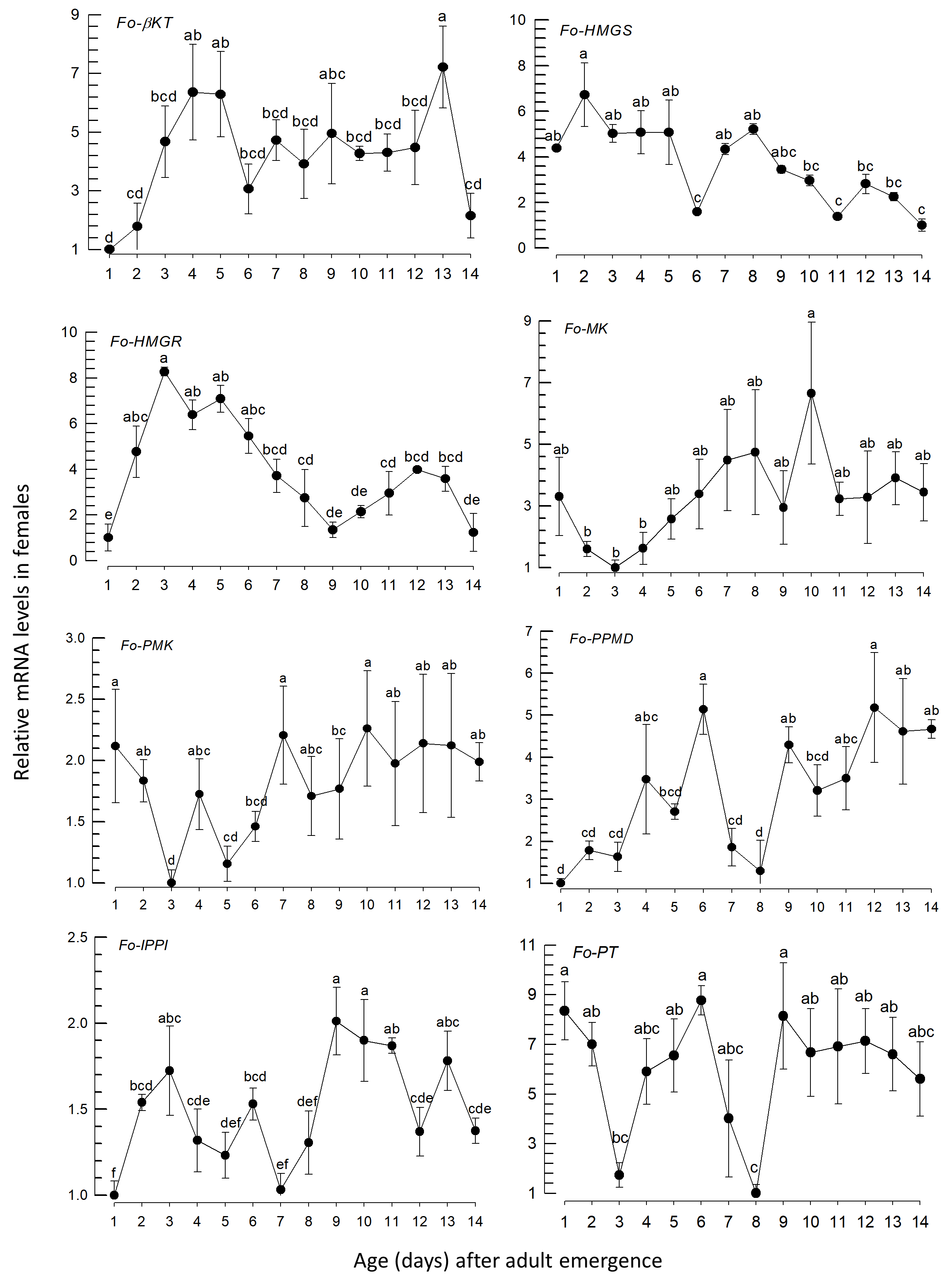


**Fig. S3**

**
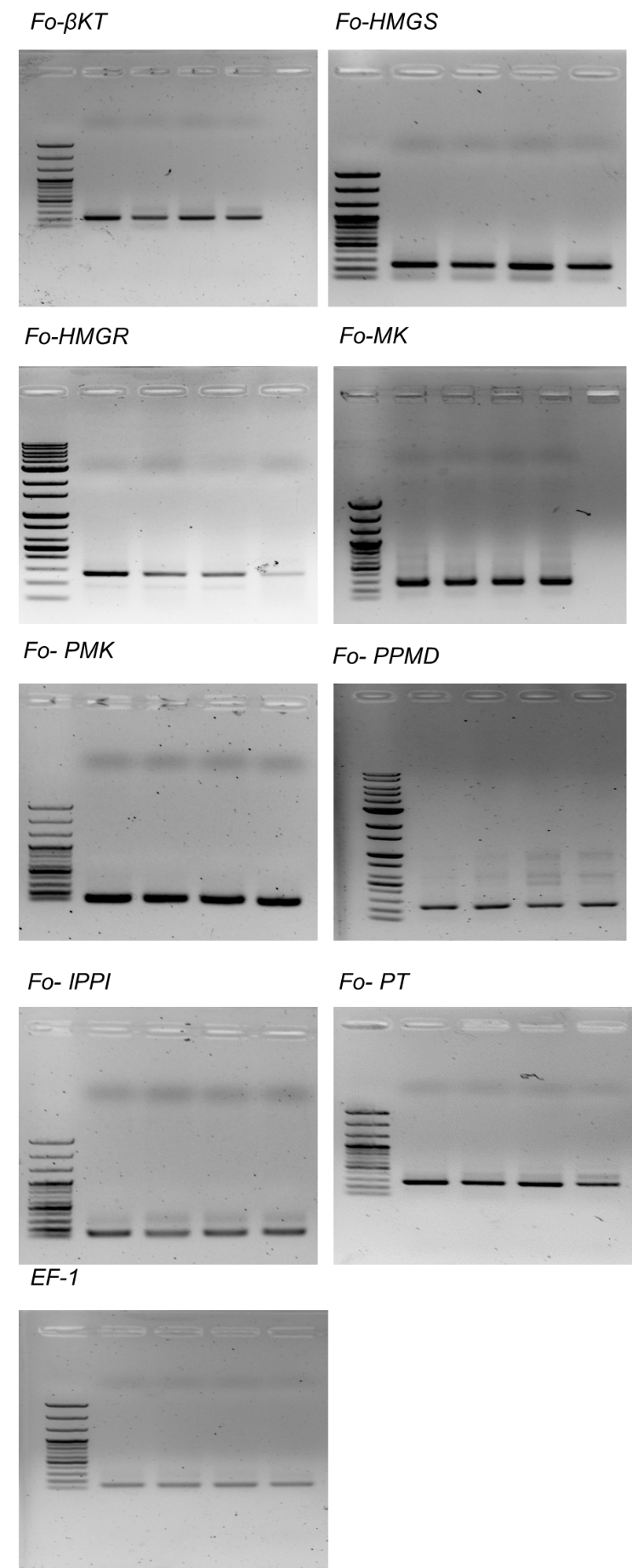
**

**Fig. S4**
